# Spatial distribution of the summer subsurface chlorophyll maximum in the North South China Sea

**DOI:** 10.1371/journal.pone.0248715

**Published:** 2021-04-07

**Authors:** Ying Chen, Hui Zhao

**Affiliations:** 1 Guangdong Ocean University, Zhanjiang, China; 2 Southern Marine Science and Engineering Guangdong Laboratory, Zhuhai, China; Guangzhou University, CHINA

## Abstract

Based on the biological, nutrients and hydrological data in August 2018, the vertical chlorophyll a (Chl-*a*) concentration profiles and the relationship among surface Chl-*a* (Chl-*a*_(0)_) concentration, maximum Chl-*a* (Chl-*a*_(m)_) concentration and depth-integrated Chl-*a* (Chl-*a*_(int)_) concentration were studied in the Northern South China Sea (NSCS). The results indicate that there are 4 different patterns in the vertical Chl-*a* profiles in the NSCS: (i) Chl-*a* increases with depth from the surface (e.g. station 1); (ii) there exists subsurface chlorophyll maximum (SCM), with low Chl-*a* on the surface and at the bottom layers respectively (e.g. station 5); (iii) there is no SCM, only with high Chl-*a* on the surface and in the bottom (e.g. station 14); (iv) the 4^th^ pattern is similar to (ii), with the higher Chl-*a*_(0)_ (e.g. station 28). The SCM is observed at 95% stations in the NSCS and is not detected only at a few stations near the Pearl River (PR) estuary. These patterns are mainly regulated by alternative limitation of nutrients and light from the surface to the bottom of euphotic layer. For the pattern 1 (e.g. station 1), light is not a limited factor, and Chl-*a* and nutrients increase with depth. The pattern 2 (e.g. station 5) exists with the limitation of surface nutrients in offshore region. The nutrients increases with depth and the nutrients limitation turns to light limitation gradually from surface to bottom. And the SCM appears in the layer which need of the light and nutrients is roughly equivalent. Compared with that the offshore SCM, the nutrients for the pattern 3 (e.g. station 14) are rich on the surface with nutrients concentration and light irradiance. Therefore, it is seawater intrusion from the bottom that brings the higher nutrients concentration. The reason for the high Chl-*a*_(0)_ on the pattern 4 (e.g. station 28) is terrestrial matter from the nearshore. High correlation (R^2^ = 0.5206, p<0.01) between the depth of SCM (Depth_(m)_) and Chl-*a*_(0)_ indicates that the SCM depth is regulated by light masking effect of surface phytoplankton, generally with shallow nutriclines and fast light attenuation for high Chl-*a*_(0)_ and vice versa low Chl-*a*_(0)_ brings deeper nutriclines and light attenuate slowly with less shading effect. Further research results shows that Chl-*a*_(int)_ and Chl-*a*_(m)_ have a good correlation(R^2^ = 0.6397, p<0.01). However, the correlation between Chl-*a*_(int)_ and Chl-*a*_(0)_ is relative weak (R^2^ = 0.3202, p<0.01). That could be attributed to the availability of nutrients playing an important role in growth of phytoplankton, with high nutrients at upper euphotic layers for the stations with high Chl-*a*_(0)_.

## 1. Introduction

The South China Sea (SCS) is the largest semi-enclosed deep-water marginal sea in the Northwest Pacific, with a unique geographical location [1–4]. It is an oligotrophic sea area with an overall area of about 3.5×10^6^ km^2^, and most of the upper layer belongs to the tropical sea area with sufficient sunlight [5, 6]. The Northern South China Sea (NSCS) has not only nutrient-rich salinity-low estuaries such as the Pearl River (PR) Estuary and coastal zones, but also oligotrophic salinity-high open sea. In addition, the influence of water stratification and thermocline, as well as the differences in the vertical distribution of light, temperature, salinity and nutrients, all these environmental factors determine that the NSCS has extremely complex ecological characteristics.

Marine phytoplankton, as one primary producer of marine organic matter, plays an important role in the carbon cycle and energy flow of the marine ecosystem [7, 8]. The concentration of chlorophyll a (Chl-*a*) is an important indicator of the marine phytoplankton biomass, and its spatial-temporal variation regulates marine primary productivity. Nutrients and Chl-*a* are two important environmental parameters which are paid more and more attention recently, because they have important scientific significance for reflecting the environmental characteristics, photosynthesis conditions, biogenic elements, and eutrophication of water bodies [9]. Moreover, research on the environmental effects of marine phytoplankton has important implications for issues such as marine fisheries, sea-air interaction, and global warming.

The subsurface chlorophyll maximum (SCM) is the phenomenon that the maximum Chl-*a* concentration occurs at a certain depth below the sea surface (i.e, subsurface) in stratified upper oceans, and the layers of SCM are nearly ubiquitous in stratified surface waters [10]. The formation of SCM is that phytoplankton adapts to low light conditions with relatively high concentrations of nutrients, which leads to increased phytoplankton biomass at these depths [11]. The vertical Chl-*a* distribution is irregular, and the SCM exists all year round in most tropical and subtropical areas, as well as in oceans and coastal waters in summer [12].

At present, there are many studies on the spatial distribution of surface Chl-*a* in the SCS, such as high Chl-*a* are located in the coastal zones with strong upwelling [13–15]. Ekman pumping and offshore Ekman transport caused by eastern Asian monsoons resulted in high Chl-*a* in the offshore regions off southeastern Vietnam and northwestern Luzon island in the SCS [16, 17]. Impacts of coastal upwelling and river discharge caused by southwest monsoon in summer induced phytoplankton biomass higher in the NSCS [18, 19]. Zhao et al. [20] analyzes the interannual variability of Chl-*a* in the SCS and elucidated possible impact of upwelling and offshore currents on Chl-*a* changes. Chen et al. [21] studies the relationship between nutrients and Chl-*a* in spring and the result shows clearly that nitrogen (N) limits phytoplankton growth in early spring in the SCS where N_2_-fixation contributes little to new production in this oligotrophic system. With the development of remote sensing technology, the use of satellite remote sensing technology to detect surface Chl-*a* has the advantages of fast detection speed, low cost and large-scale synchronous measurement compared with conventional methods [22, 23]. The previous studies show that, 30%~70% of primary water productivity is contributed by this subsurface chlorophyll maximum layer [24–26].

But the SCM can not be monitored by satellite sensors. Few studies in the SCS explored the vertical Chl-*a* distribution and the relationship between surface Chl-*a* (Chl-*a*_(0)_), maximum Chl-*a* (Chl-*a*_(m)_) and depth-integrated Chl-*a* (Chl-*a*_(int)_). Therefore, based on the information of SCM obtained by in-situ observations in the SCS, it is necessary to study the vertical distribution and main controlling factors of SCM in the NSCS, which is of great significance to elucidate the regulation mechanism of Chl-*a*, and can provide important ecological parameters for the study of marine biogeochemical model. In addition, studying the spatial-temporal distribution characteristics and influencing factors are also conducive to improving the calculation accuracy of Chl-*a* concentration in the euphotic layer, providing a theoretical basis for the evaluation of marine fisheries [27] and deepening the understanding of the global marine carbon cycle [28, 29].

## 2. Materials and methods

### 2.1 Study area

**Fig 1:**
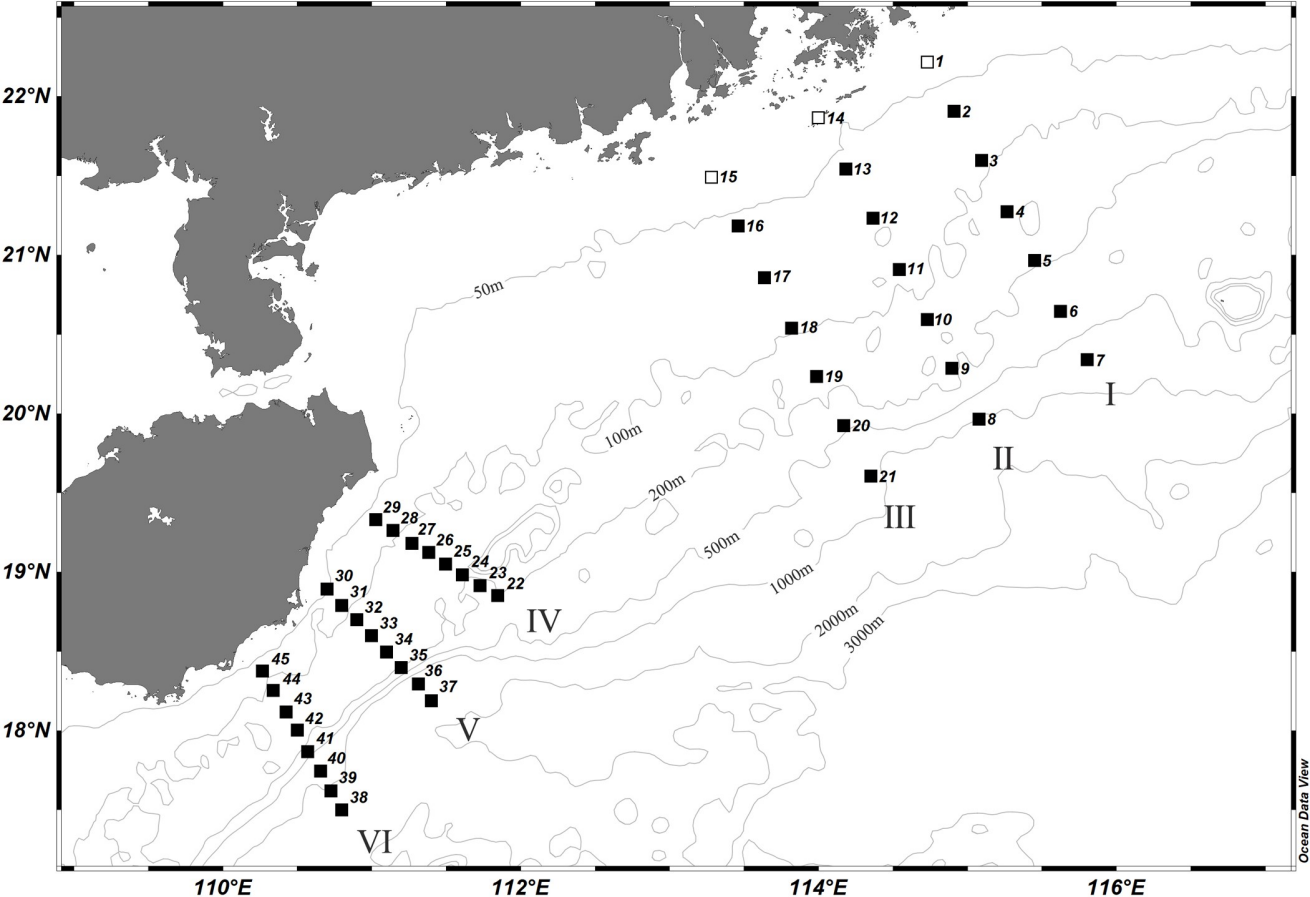
The study area. (Dark symbols mark stations where SCM was observed.).

A total of 45 stations are conducted in the NSCS during the comprehensive scientific survey of the South China Sea Institute of Oceanology, Chinese Academy of Sciences, aboard the RV "Shiyan 3" ship in August, 2018. The surveyed sea area is from the PR estuary to the southeast of Hainan (Fig 1), within the range of 17.0°N-22.5°N, 109.0°E -117.0°E. There are 6 survey sections (sections Ⅰ, Ⅱ, Ⅲ, Ⅳ, Ⅴ, Ⅵ).

### 2.2 Data and methods

The investigation was carried out regarding the Specification of Oceanographic Investigation issued by the State Oceanic Administration, China. Since this work only took seawater from specific stations in the NSCS for detection and analysis, there was no requirement for ethical approval of sampling protocols. The field studies did not involve endangered or protected species. Different water layers samples of all stations were collected at different water depths using rosette sampler carrying ten 12-dm^3^ Niskin bottles (General Oceanics, Inc., Miami, FL, USA) at the different sampling stations on the cruise route in August (summer) in 2018. The vertical profiles of water temperature, salinity, depth, photosynthetically active radiation (PAR) and fluorescence were obtained using a CTD (Conductivity, Temperature, Depth) Profiler (Sea Bird 911 Plus).

The environment parameters including nutrients (NO_3_-N, NO_2_-N, PO_4_-P and NH_4_-N) and Chl-*a* were measured based on “The specialties for oceanography survey” [30]. Dissolved inorganic nitrogen (DIN) was the sum of NO_2_-N, NO_3_-N and NH_4_-N. The detail information of the above-mentioned parameters and analytical methods were shown in the reference [31]. The Chl-*a* data files (Fig 7B) were freely available from the GlobColour dataset (https://hermes.acri.fr/).

### 2.3 Data analysis

There is a good correlation (R^2^ = 0.7407, p<0.01) between in-situ Chl-*a* and CTD-derived fluorescence. Based on the above linear relation, the CTD-derived fluorescence was processed into Chl-*a* concentrations for further analysis. By the linear interpolation method, the CTD-derived Chl-*a* concentrations at all stations were interpolated to the values with interval of 1 meter, and then depth-integrated CTD-derived Chl-*a* (i.e. depth-integrated Chl-*a* concentration) was calculated as follows:

$$Chl-a_{\left(int\right)}=\sum_{i=1}^{n-1}\frac{Chl-a_{\left(i\right)}+Chl-a_{\left(i+1\right)}}{2}\times (D_{i+1}-D_{i})$$

Where Chl-*a*_(int)_ is the depth-integrated Chl-*a* concentration in water column (mg·m^-2^) (here Chl-*a*_(int)_ is calculated from surface to the bottom or the 200-m layer if the water depth is over 200 m), Chl-*a*_(i)_ is the concentration of chlorophyll in the layer (i) (mg·m^-3^), D_i_ is the sampling depth in layer i (m), n is the sampling layer.

All the above-mentioned data analysis methods were performed in the software Origin, Ocean Data View and MATLAB.

## 3. Results

### 3.1 Vertical profiles of Chl-*a*、temperature and DIN in NSCS

**Fig 2:**
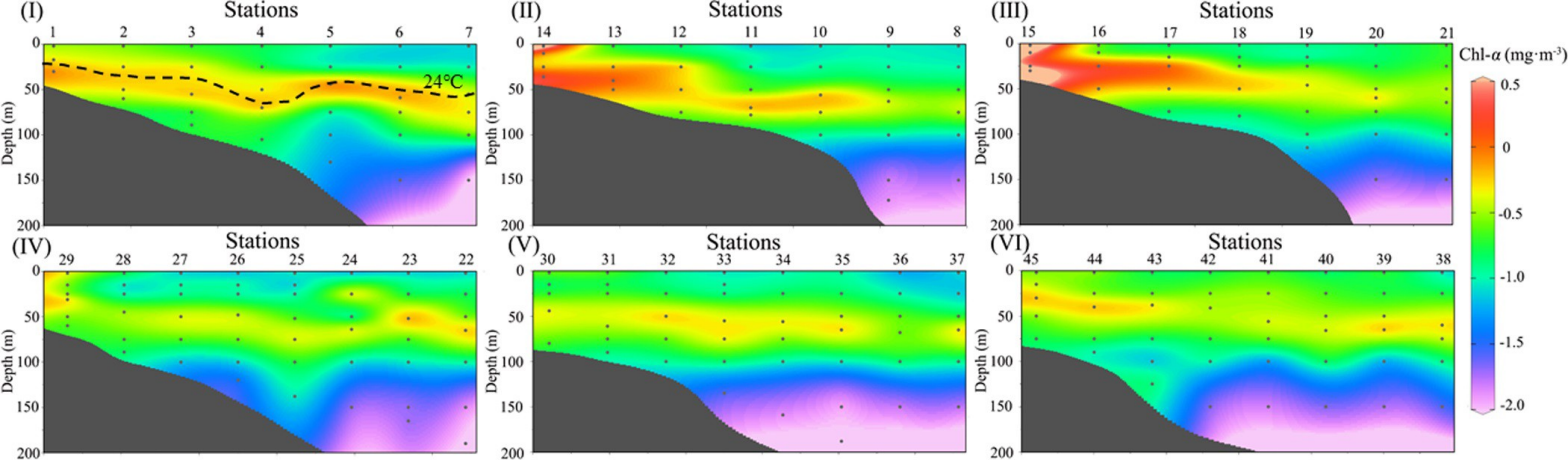
Vertical Chl-*a* distribution along the six sections. (The Chl-*a* is processed by logarithm with base of 10).

In the Fig 2, it shows the Chl-*a* profiles along with the six sections. The Chl-*a*_(0)_ at each section decreases from nearshore to offshore. Furthermore, the SCM depth presents a decreasing trend from nearshore to offshore along with these sections. In addition, the SCM layer at station 5 at the depth of 50 meters is elevated on section I. Sections II and III shows that there is no existence of the SCM layer at the stations near the PR esturay with decreasing surface Chl-*a* and increasing the SCM layer from the river estuary to offshore stations. Station 14 and station 15 in sections II and III near the PR estuary are greatly affected by terrestrial input, and Chl-*a* shows a high value on the surface and at the bottom. Other stations have a SCM phenomenon. The patterns of Chl-*a* in sections IV, V, and VI are similar, and SCM lies between 40–50 meters. In six sections, the higher Chl-*a* in the nearshore water is due to the shallower water depth and potential input of terrigenous materials, while the water depths at the offshore stations exceed mostly the depths of the euphotic layer, where the Chl-*a* is low, with the lowest Chl-*a* value near 200 meters layer at the above stations.

**Fig 3:**
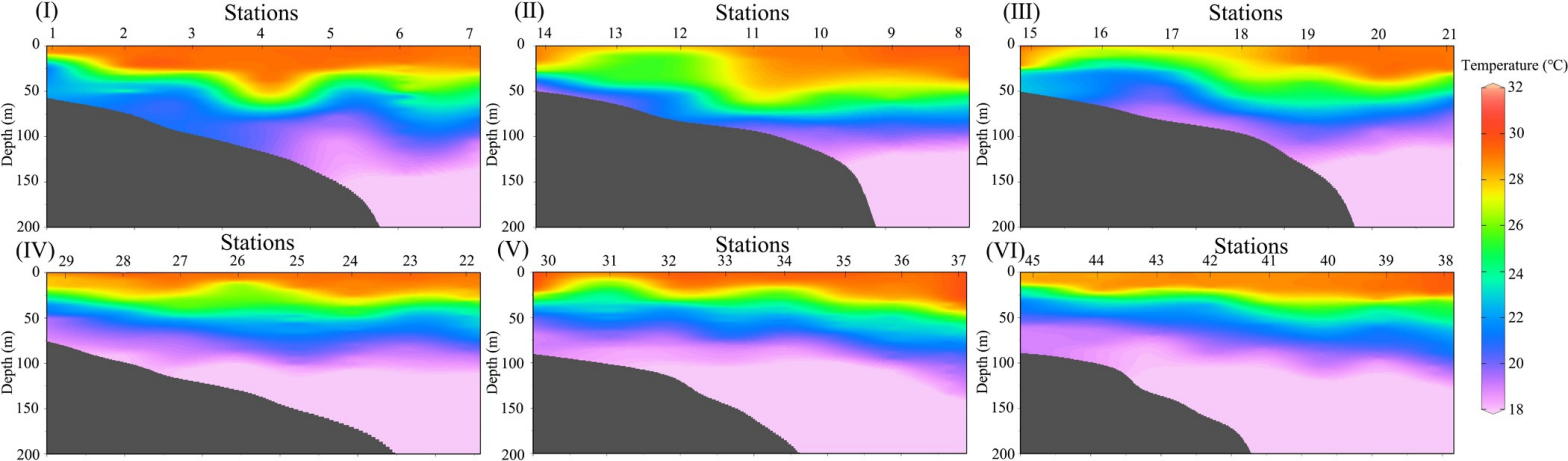
Vertical profiles of temperature along six sections.

In the Fig 3, it shows the temperature profiles along with the 6 sections. The thermocline depth of these 6 sections is the lowest in the nearshore, with the increasing thermocline depth gradually toward offshore increases. The depth of the 24^0^C isotherm in stations 1–7 (section I) is coincided with the depth of SCM in Fig 2 (section Ⅰ). In the range of 20°C -27.5°C, station 5 has a rising trend compared with station 4 and 6 in section I. It is speculated that there is an upwelling in station 5 and a downwelling in station of 4 and 6. The temperature sections II-III show the surface temperature in the nearshore is lower than the offshore surface temperature, due to influence of the PR diluted water on nearshore stations. The temperature sections IV Ⅴ and VI has the similar characteristics that the depth of the thermoclines is the lowest in the nearshore and the depth increases slightly towards the open sea.

**Fig 4:**
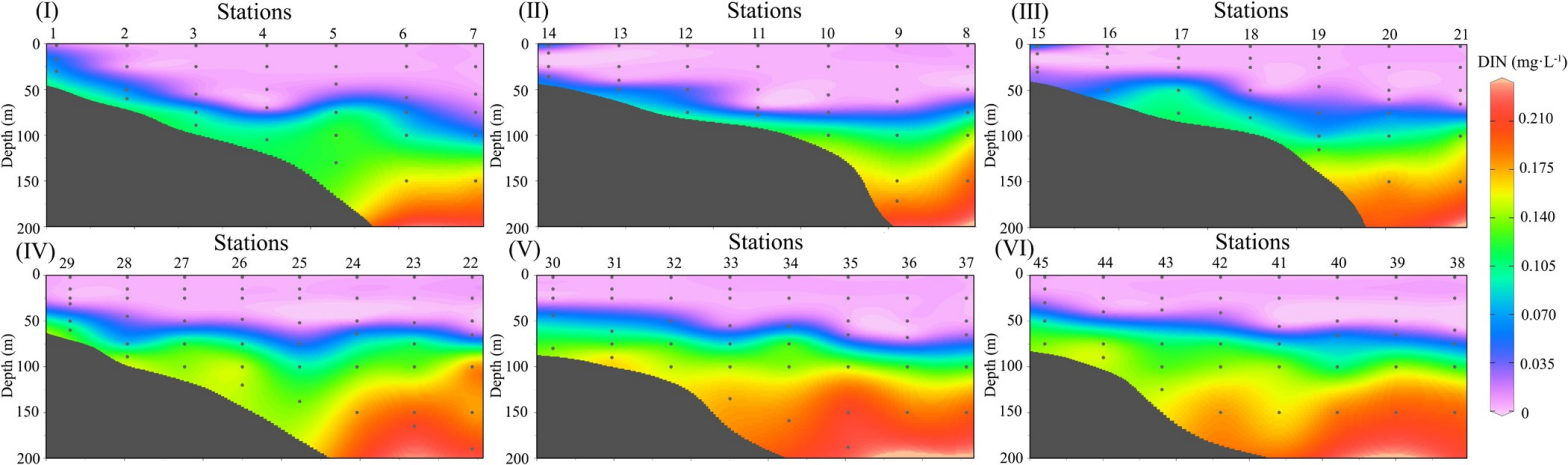
Vertical profiles of DIN along the six sections shown in the Fig 1.

In the Fig 4, it shows the DIN distribution along with the 6 sections. The DIN is low on the surface and become gradually higher below 50 meters, reaching the maximum value at the bottom. This is because that the main source of DIN in the offshore is the DIN-intensive area induced by decomposition of organic matter and no photosynthesis at the bottom, and the existence of the thermocline prevents the vertical diffusion of nutrients. At the same time, the growth of phytoplankton also needs consume a lot of nutrients in the shallow euphotic layers, so low DIN in the offshore waters is above 50 meters. The nearshore surface on section I, II and III has higher DIN, which is mainly due to the increase in nutrients brought by rivers entering the sea along the coast.

### 3.2 Chl-*a* distribution in typical stations

Justic et al. [32] proposed one standard of nutrient limitation based on the ratio of Redfield [33]: (1) When Nitrogen (N): Phosphorus (P) >22 and Silicon (Si): P>22, P is the limited factor; (2) When N: P<10 and Si:N>1, N is the limited factor; (3) When Si:P<10 and Si:N<1, Si is the limited factor. The results show that station 1 has no restrictive factor, station14 and station15 are P-limitation, and the remaining stations are N-limitation. There is no Si limitation among all stations. Therefore, the silicon limitation is not considered. Otherwise, temperature and salinity are also important factors for phytoplankton growth.

**Fig 5:**
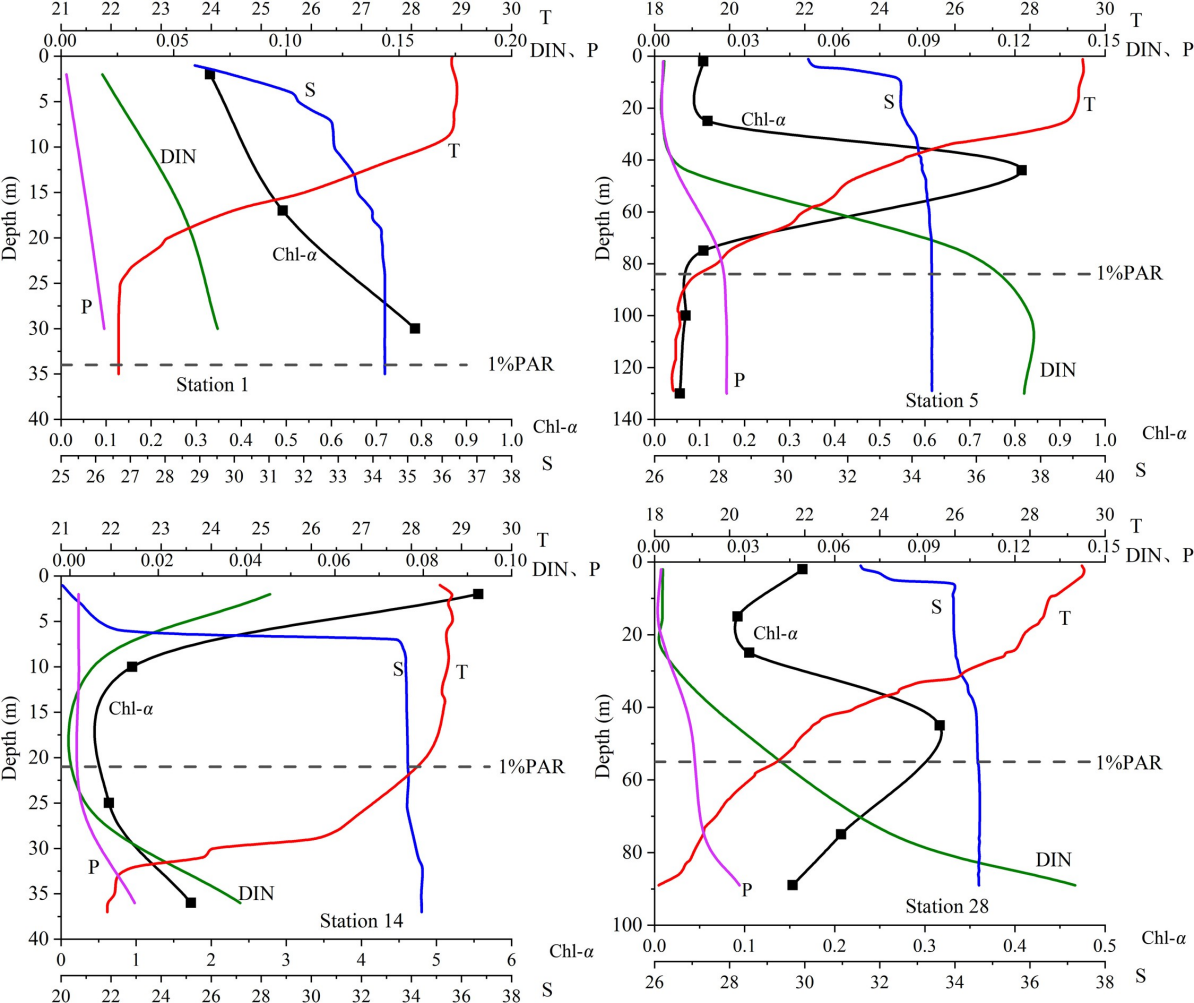
Types of vertical profiles of Chl-*а* (Chl-*a*, mg·m^-3^), DIN (DIN, mg·L^-1^), Phosphorus(P, mg·L^-1^), Temperature(T,°C) and Salinity(S,‰) along the typical station. The horizontal dashed lines is the depths with 1% PAR, representing the depth of euphotic layers.

Among the 45 stations, more than 95% of the stations have SCM, and the vertical Chl-*a* profiles can be subdivided into 4 patterns (Fig 5) according to their vertical changes of Chl-*a* with depth. The pattern 1 of vertical Chl-*a* distribution is that Chl-*a* is the lowest on the surface layer. The Chl-*a* increases with depth from the surface, and the maximum appears at the bottom (e.g. station 1 in Fig 5). Due to the station 1 in the nearshore, the nutrients are rich on the surface. The Chl-*a* range is 0.33–0.79 mg·m^-3^, and the highest Chl-*a* appears at the bottom. The vertical Chl-*a* distribution is represented by station 5 in Fig 5 (named by the pattern 2) accounts for more than 90%. The pattern 2 (e.g. station 5 in Fig 5) of vertical Chl-*a* distribution is investigated by the SCM with lower values on the surface and at the sea bottom. As an example, the Chl-*a* range at station 5 is from 0.06–0.82 mg·m^-3^, and the depth of SCM is at 44 meters. With the increase of depth, the Chl-*a* firstly decreases to a certain depth and then begins to increase until there is a maximum peak value in the subsurface. The pattern 3 represented by the station14 (Fig 5) is located in the diluted water area of the PR estuary. The vertical Chl-*a* distribution presents as the trend of inverted " U " shape with an opening to the right obviously. The changing trend of DIN is synchronized with the change of Chl-*a*, and higher DIN and Chl-*a* is on the fresh surface. The Chl-*a* and the DIN decrease gradually, to the minimum values at the depth of ~15 meters, and then Chl-*a* increases gradually from the depth to the bottom synchronously with DIN. The pattern 4 (e.g. station 28 in Fig 5) presentes one ‘S’ shape with relatively high surface Chl-*a* and SCM. It is different from pattern 2 that the Chl-*a* decreases first gradually at the upper layer of 0–20 meters, and then the Chl-*a* increases with depth between 20–48 meters, and then decreases with the depth below 40 meters.

### 3.3 The relationship between Chl-*a*_(0)_、Chl-*a*_(m)_ and Chl-*a*_(int)_

**Fig 6:**
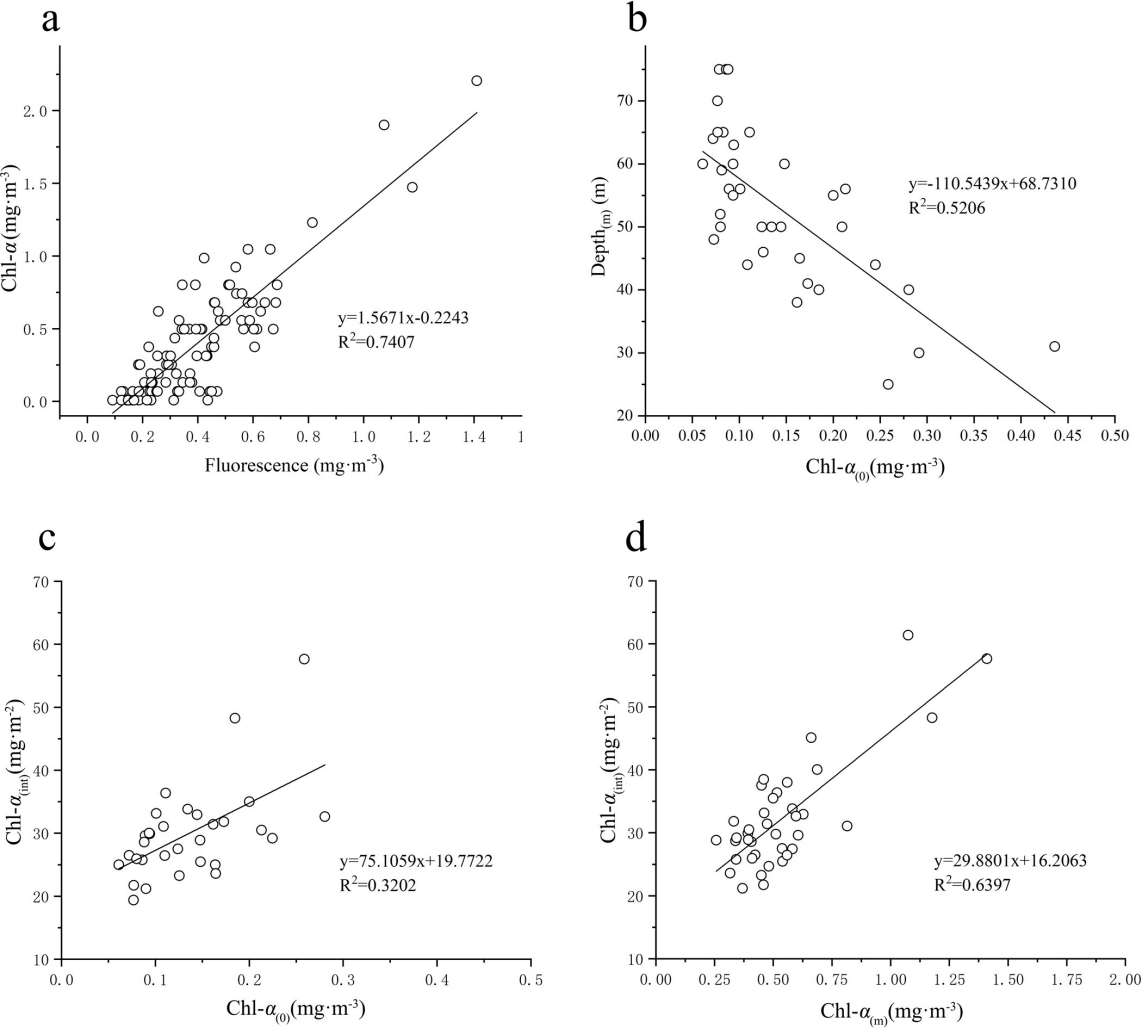
a: The relationship between Chl-*a* (mg·m^-3^) and Fluorescence (mg·m^-3^), b: The relationship between Chl-*a*_(0)_ (mg·m^-3^) and Depth (m), c: The relationship between Chl-*a*_(0)_ (mg·m^-3^) and Chl-*a*_(int)_ (mg·m^-2^), d: The relationship between Chl-*a*_(m)_ (mg·m^-3^) and Chl-*a*_(int)_ (mg·m^-2^).

The relationship between fluorescence and Chl-*a* measured is analyzed in the lab (Fig 6A). Their liner relationship (R^2^ = 0.7407, p<0.01) shows that fluorescence can be used to estimate Chl-*a*. Therefore, we use the fluorescence-derived Chl-*a* to calculate the Chl-*a*_(int)_. In the Fig 6B, the relationship among Chl-*a*_(0)_ and Depth_(m)_ (R^2^ = 0.5206, p<0.01), and the correlation with the Chl-*a*_(0)_, Chl-*a*_(m)_ and Chl-*a*_(int)_ (Fig 6C and 6D) shows that the Chl-*a*_(m)_ has a good linear relationship with the Chl-*a*_(int)_ (R^2^ = 0.6397, p<0.01) (Fig 6D), however, the correlation between Chl-*a*_(0)_ and Chl-*a*_(int)_ is relatively weak (R^2^ = 0.3202, p<0.01) (Fig 6C).

**Fig 7:**
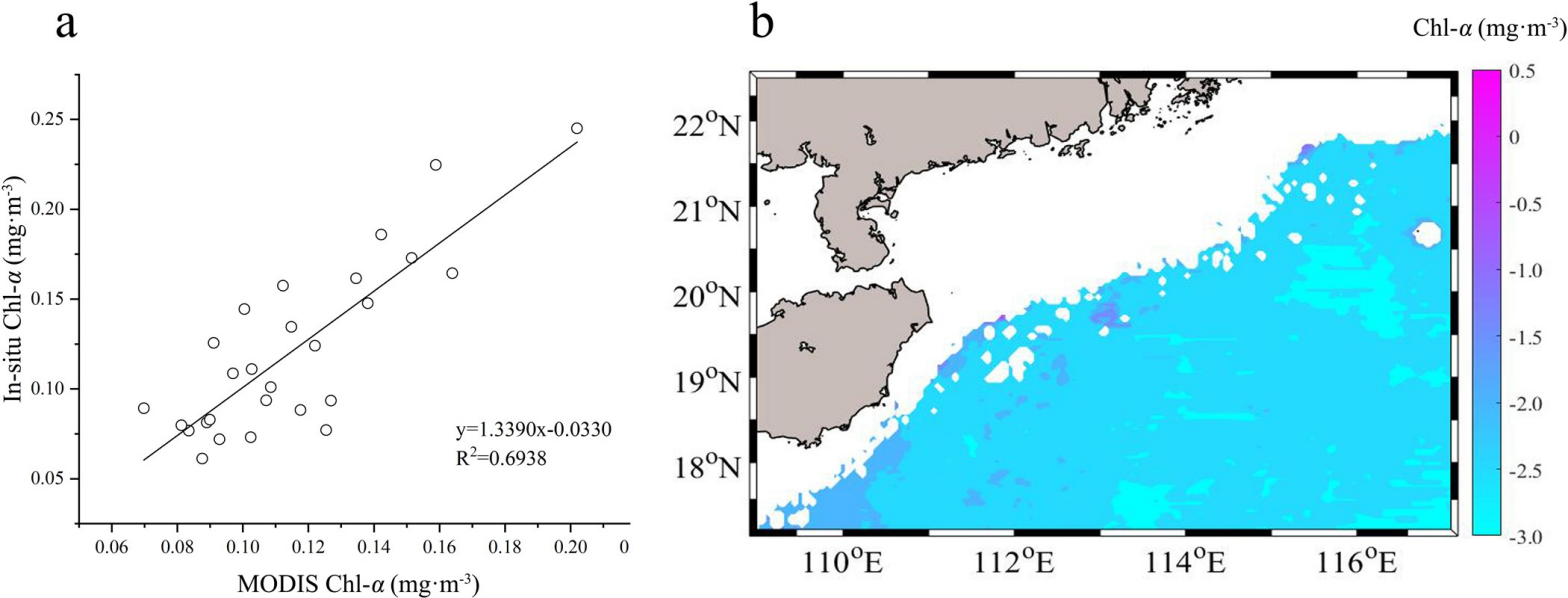
a: Relationship between In-suit Chl-*a* (mg·m^-3^) and MODIS Chl-*a* (mg·m^-3^); b: Distribution characteristics of Chl-*a* in NSCS (here we ignore the region where the depth is shallower than 100 meters to remove influence of case-2 water; The chlorophyll concentration is processed by logarithm with base of 10).

In addition, Fig 7A presentes the correlation between the In-situ Chl-*a* and the MODIS-derived Chl-*a* data. The trendency of the two sets of data shows good consistency (R^2^ = 0.6938, p<0.01), which indicates that MODIS remote sensing Chl-*a* data in the NSCS can qualitatively reflect the true Chl-*a* concentration and meets the data requirements of spatio-temporal characteristics analysis. Moreover, it shows the MODIS remote sensing Chl-*a* data in the NSCS in Fig 7B (here, the region with the water depth shallower than 100 meters is removed due to influence of case-2 water). The Chl-*a*_(0)_ in the NSCS in Fig 7B is about 9901 mg·m^-3^, so the Chl-*a*_(int)_ within 200 meters is about 13257 mg·m^-3^ from y = 1.3390x-0.0330 (Fig 7A).

## 4. Discussion

### 4.1 Preliminary study on the relationship between vertical Chl-*a* distribution and physical environment in NSCS

The SCS is the largest marginal sea in the western Pacific characterized by marked seasonality under the control of the monsoon [17]. The SCM is considered the most prominent biological feature in the vertical water column during stratified seasons. Although it is seasonal, in terms of primary productivity, the ecological significance of the SCM is equivalent to the spring phytoplankton bloom in the seas [34, 35]. It can be seen from Fig 5 that the vertical Chl-*a* profiles are significantly different, and the spatial Chl-*a* distribution is closely related to environmental conditions including temperature, salinity and nutrients. Nutrients and light are generally two of the most important limited factors for phytoplankton growth [36]. In order to explore the relationship between the vertical Chl-*a* distribution and the physical environmental conditions including nutrients, light, temperature and salinity, the environmental parameters of six sections and all four patterns of the vertical Chl-*a* distribution are analyzed in the region.

The distribution of the SCM usually varies along with the trophic status of the water layer. From the results of our research, the Depth_(m)_ is generally shallow in coastal eutrophicated areas and increases gradually towards the oligotrophic open sea. There are the SCM phenomena in the most stations of the NSCS. Moreover, phytoplankton grows mainly in the euphotic layer with relatively light radiation intensity and sufficient nutrient supply. It is worth noting that the phytoplankton population in SCM is more adaptable to relatively low light conditions with relatively high concentrations of nutrients [37]. Philips group studies in detail the relationship between plankton and light and nutrients in Okeechobee Lake [38]. It is concluded that low light have a strong effect on the community structure of plankton. Continuous low light can induce algae to reach higher population density in a short time. In other words, low light is one of the reasons for the formation of algal blooms when the light intensity is over the light compensation point [39].

When the Chl-*a*_(0)_ is low (≈ 0.1 mg m^-3^), the SCM occurs in the offshore or deep water stations. Pattern 2 basically represents the common phenomenon of the vertical Chl-*a* distribution with the SCM in offshore ocean waters. It can be seen from the section that the SCM of station 5 (Fig 2) at the depth of 50 meters has an upward trendency, when compared with station 4 and 6. Combined with the temperature (Fig 3) and DIN section (Fig 4), it is speculated that there may be have a weak upwelling of station 5 affected by the monsoon, and a weak downwelling of station 4 and 6. The upwelling could accelerate the transport of deep nutrient-rich water into the surface, which promotes the growth of phytoplankton, enhances the primary productivity of the waters, and further enriches the diversity of the whole species [40, 41]. It is also the reason for higher Chl-*a* in Station 5. The maximum Chl-*a* on station 5 appears in the subsurface. And, the vertical Chl-*a* distribution in the NSCS in summer mostly belongs to this pattern. The vertical Chl-*a* distribution of pattern 4 is similar to pattern 2, it also has a maximum Chl-*a* in the subsurface. However, affected by the nearshore, the low salinity of the upper water and the rich nutrients, higher Chl-*a* appears on the surface of pattern 4. The position of the pattern 4 is close to the coastal region with the roughly same concentration of nutrients in shallow layers (i.e. 0-20m). So the surface Chl-*a* is relatively high, compared with that of the pattern 2.

In the SCS, the water temperature distribution in the upper ocean are affected by the mixing layer. The vertical temperature and salinity variation can reflect the coastal rivers. While the nutrients carried by coastal rivers affect the growth and reproduction of phytoplankton [42]. On pattern 3, the vertical Chl-*a* distribution shows that it locates in the extension area of the PR diluted water, where covers the upper layer of the coastal station. It is obvious that the low salinity above 10 meters and halocline on the surface make the surface waters stratified. Due to the low density of the PR diluted water, it diffuses in the upper layer after the outflow of the PR estuary, and reduces the surface salinity. Moreover, the upper layer is low-salinity PR diluted water, while the bottom is surrounded by high-salinity offshore water, regarding the PR diluted water. Chl-*a* is relatively high above the halocline, however, it decreases with the salinity increasing. At coastal stations, due to the high suspended matter in diluted water of the PR and the light attenuation, the thickness of the euphotic layer is shallow. At the same time, the phytoplankton grows vigorously, because the diluted water brings rich terrestrial nutrients. High Chl-*a* can be formed in the nearshore area, in contrast, the SCM is non-existent. Because of the high concentrations of dissolved organic matter, particulate organic matter and the low transparency of water, it is not conducive to the formation of SCM in the region. Pattern 1 provides the necessary conditions for the growth of phytoplankton with the shallow water depth and rich light, Chl-*a* and nutrients increase with depth. In addition, the unique wind system in the SCS and its interaction with terrain largely determine the dynamics of the region [43]. Monsoon is the most important factor affecting regional circulation and dynamics, and also affects the concentration level of Chl-*a*. Affected by the southwest monsoon prevailing in the SCS in summer, a strong vertical mixing is caused by wind stress and convection. Besides, an upwelling affected by Coriolis force is formed along with the coast, and the high-salty water is in the offshore wedges from the bottom. This is the reason why the Chl-*a* distribution of stations 1 and 14 are high at the bottom and low in the middle.

### 4.2 Responses of Chl-*a*_(int)_ to Chl-*a*_(0)_ and Chl-*a*_(m)_

In general, the suitable or excessive temperature and nutrients are another primary reason for species succession, proliferation and aggregation of phytoplankton. The species and biomass of phytoplankton can change and even “crazy growth” occur, leading to phytoplankton bloom. The occurrence of phytoplankton bloom involves in a series of complex processes including movement and development processes from the emergence, proliferation, aggregation to eutrophication of phytoplankton [44]. These processes are roughly divided into three stages. (i) The high growth of initial nutrient concentration leads to the rapid-proliferation of phytoplankton; (ii) A large number of nutrients would always be consumed in the rapid proliferation of phytoplankton, resulting in nutrient concentration decreasing; (iii) Proliferation and massive dead of phytoplankton are affected by the continuous decline in nutrient concentrations [44]. In addition to the direct relationship with the occurrence of water eutrophication and the large input of alien nutrients, the destruction of biological community structure in water leads to the decrease of self-purification capacity, which is also an important reason for accelerating water eutrophication.

Light is one of the main ecological factors of water ecosystem. The total photosynthesis rate increases roughly with the light intensity within a certain range, and the photosynthesis rate generally increases no longer after the light saturation point under no nutrient-limited conditions [45]. Under the same light conditions, especially in the environment of low light intensity, such as in the period of insufficient light in spring, the increasing phytoplankton in eutrophic layer would lead to serious self-shading effect, which influences the subsurface Chl-a growth environment by altering the penetration of light. When nutrients is saturated below the surface layers, higher phytoplankton biomass is in the shallower layer. Then the faster light attenuation due to the shading effect of phytoplankton become the limiting factor of the growth of phytoplankton. It can be seen from Fig 6 that the Chl-*a*_(0)_ (R^2^ = 0.3202, p<0.01) and Chl-*a*_(m)_ (R^2^ = 0.6397, p<0.01) are positively correlated with the Chl-*a*_(int)_, respectively. However, the correlation between Chl-*a*_(int)_ and Chl-*a*_(0)_ is relatively weak, compared with that between Chl-*a*_(int)_ and Chl-*a*_(m)_. When the higher Chl-*a*_(0)_ on surface layers, it would result in the stronger shading effect. This makes the light radiation easier be insufficient in the vertical direction, and limites the growth of phytoplankton in subsurface layers. A negative correlation between the Chl-*a*_(0)_ and Depth_(m)_ (R^2^ = 0.5206, p<0.01) also indicates that the shading effect of high Chl-*a*_(0)_ leads to decrease of the Depth_(m)_. Otherwise, the rapid growth of phytoplankton in rich-light shallower layers consumes a large number of nutrients, resulting in the decline of nutrients. The growth of phytoplankton is restricted again in the upper ocean. Previous studies [46–48] indicates generally the offshore SCS was oligotrophic in the upper water. Although the higher Chl-*a*_(0)_ may exert positive contribution on the Chl-*a*_(int)_, the higher Chl-*a* also induces the more significant shading effect with negative influence to the Chl-*a*_(int)_. Therefore, the correlation (R^2^ = 0.3202, p<0.01) is relatively weak between Chl-*a*_(int)_ and Chl-*a*_(0)_, compared with that between Chl-*a*_(int)_ and Chl-*a*_(m)_. The results of Gong group indicates that the depth of SCM with higher Chl-*a*_(m)_ are often shallow [10]. Similar to our study, there is significant negative correlation between Chl-*a*_(m)_ and Depth_(m)_, which may be regulated by exponential attenuation of light as well as nutrients. The higher Chl-*a*_(m)_ follows the shallower Depth_(m)_. The Chl-*a*_(m)_ appears generally the place where there are relative high nutrients with adequate lightone better compromise between light and nutrients, therefore the Chl-*a*_(int)_ presents a good correlation with the Chl-*a*_(m)_.

### 4.3 Potential formation mechanism of SCM in the SCS

The formation and maintenance of the SCM includes the physical and biological processes, and changes with the water nutritional status and hydrodynamic conditions [49]. The formation of the SCM have been confirmed and improved with the development of observation techniques and the expansion of study areas. The contributions of different mechanisms are mainly controlled by the vertical nutrient flux and light availability. It is important to identify the distribution and dynamics of the SCM to determine the ecological significance of the SCM in ecosystem. The contribution of different mechanisms may differ widely in different marine systems.

It is believe that in far coast of the SCS, the upper water has a low nutrient and stronger light for phytoplankton growth. The phytoplankton is inhibited by both nutrition and light, resulting in low Chl-*a*. As the depth increases, the light decreases and the nutrients increase to making SCM appear in the subsurface layer, which is conducive to the growth of phytoplankton. The depth of the SCM indicates that this position is the relatively suitable nutrient concentration and light conditions for phytoplankton growth in the vertical direction, but it does not mean that this is the most suitable nutrient and light conditions, just relative to the vertical water column in these positions. The study area, it is a oligotrophic sea areas with low Chl-*a*_(0)_. With the increase of subsurface nutrients and the decrease of light, the maximum Chl-*a* usually appears in the subsurface. The formation and maintenance mechanisms of SCM in different sea areas are different, however, it is generally believed that the most common reason for the formation of SCM is the rapid growth of phytoplankton in the subsurface with suitable light and nutrient conditions. In the offshore in the SCS, with the low Chl-*a* in the upper water, the growth of phytoplankton depletes available nutrients quickly in the upper water column. The SCM gradually migrates to a deeper place, depressing the nutrients until it approaches the compensation depth (reaching the point of basic productivity and respiratory balance). The SCM continues to grow actively at this depth to acclimate to the low light conditions of the subsurface [50]. With respect to two opposing gradients (light from above and nutrients from below), the SCM represents the optimal depth for phytoplankton growth.

## 5. Conclusions

Based on the in-suit data of Chl-*a*, nutrients, temperature and salinity in the NSCS in summer, the vertical Chl-*a* distribution and the relationship between Chl-*a*_(0)_, Chl-*a*_(m)_ and Chl-*a*_(int)_ are investigated. The studies show that the distribution of Chl-*a* is associated with temperature, salinity and nutrients. The SCM exists in most of the offshore NSCS (except those in the nearshore). The results are attributed to the fact that nutrient limitations in shallow layers are gradually converted to light limitations with increase of the depth. The SCM appears in the subsurface layer where there are relative high nutrients and relative sufficient light. In addition, the Chl-*a*_(m)_ has a good correlation with Chl-*a*_(int)_, while the correlation between the Chl-*a*_(0)_ and the Chl-*a*_(int)_ are weak. The shading effect of higher Chl-*a*_(0)_ reduces Depth_(m)_ and limits phytoplankton growth. In this paper, the results may help to improve our understanding of phytoplankton growth dynamics and global carbon cycle. By exploring the relationship between Chl-*a*_(int)_ and Chl-*a*_(0)_, the relationship is established, and the overall Chl-*a* of the sea water in the SCSN can be obtained entirely through the surface Chl-*a*. The in-suit Chl-*a* from cruises has long sampling period, less sampling stations, large sampling interval, sampling environment can not be estimated and other unfavorable factors. The remote sensing data has the advantages of fast detection speed, low cost and large-scale synchronous measurement, so the remote sensing data of Chl-*a* can be used to obtain the overall Chl-*a* of the SCS or other sea areas entirely. Using remote sensing technology to retrieve global ocean Chl-*a* timely and accurately is of great significance for scientific research. In addition, the study on its spatio-temporal distribution and influencing factors is conducive to improving the calculation accuracy of Chl-*a* in the euphotic layer. It also deepens the understanding of the vertical and horizontal distribution characteristics of the SCM in the SCS, and its formation and change mechanism.

## Supporting information

S1 Fig The study area. (Dark symbols mark stations where SCM was observed).
(PDF)

S2 Fig Vertical Chl-a distribution along the six sections. (The Chl-a is processed by logarithm with base of 10).
(PDF)

S3 Fig Vertical profiles of temperature along six sections. (PDF)

S4 Fig Vertical profiles of DIN along the six sections shown in the Fig 1. (PDF)

S5 Fig Types of vertical profiles of Chl-а (Chl-a, mg·m^-3^), DIN (DIN, mg·L^-1^), Phosphorus(P, mg·L^-1^), Temperature(T,°C) and Salinity(S,‰) along the typical station. (PDF)

S6 Fig a: The relationship between Chl-a (mg·m^-3^) and Fluorescence (mg·m^-3^), b: The relationship between Chl-a_(0)_ (mg·m^-3^) and Depth (m), c: The relationship between Chl-a_(0)_ (mg·m^-3^) and Chl-a_(int)_ (mg·m^-2^), d: The relationship between Chl-a_(m)_ (mg·m^-3^) and Chl-a_(int)_ (mg·m^-2^).
(PDF)

S7 Fig a: Relationship between In-suit Chl-a (mg·m^-3^) and MODIS Chl-a (mg·m^-3^); b: Distribution characteristics of Chl-a in NSCS.
(PDF)
