# Supplementary material for: Spatial distribution of the summer subsurface chlorophyll maximum in the North South China Sea
Source: PLoS One. 2021 Apr 7;16(4):e0248715. doi: 10.1371/journal.pone.0248715 (PMC8026054; doi:10.1371/journal.pone.0248715)
Supplement: S1 Fig — (Dark symbols mark stations where SCM was observed). (PDF) [file pone.0248715.s001.pdf]

S1 Fig . The study area. (Dark symbols mark stations where SCM was observed.)

Station	lon	lat	Station	lon	lat
1	114.73	22.22	2	114.91	21.9
3	115.09	21.59	4	115.27	21.27
5	115.45	20.96	6	115.63	20.64
7	115.81	20.33	8	115.08	19.96
9	114.9	20.28	10	114.72	20.59
11	114.54	20.91	12	114.36	21.23
13	114.18	21.54	14	114	21.86
15	113.28	21.49	16	113.46	21.18
17	113.64	20.86	18	113.82	20.54
19	113.99	20.23	20	114.17	19.92
21	114.35	19.6	22	111.85	18.85
23	111.731	18.919	24	111.613	18.988
25	111.494	19.056	26	111.375	19.125
27	111.256	19.194	28	111.138	19.263
29	111.019	19.331	30	110.7	18.9
31	110.8	18.8	32	110.9	18.7
33	111	18.6	34	111.1	18.5
35	111.2	18.4	36	111.3	18.3
37	111.4	18.2	38	110.8	17.5
39	110.725	17.625	40	110.65	17.75
41	110.575	17.875	42	110.5	18
43	110.425	18.125	44	110.35	18.25
45	110.275	18.375			
