# Supplementary material for: Spatial distribution of the summer subsurface chlorophyll maximum in the North South China Sea
Source: PLoS One. 2021 Apr 7;16(4):e0248715. doi: 10.1371/journal.pone.0248715 (PMC8026054; doi:10.1371/journal.pone.0248715)
Supplement: S2 Fig — (The Chl-a is processed by logarithm with base of 10). (PDF) [file pone.0248715.s002.pdf]

S2 Fig . Vertical Chl-a distribution along the six sections. (The Chl-a is processed by logarithm with base of 10)

Station	Depth（m）	Chla(μg/L)	Station	Depth（m）	Chla(μg/L)
1	2	0.33	25	75	0.41
1	17	0.49	25	100	0.18
1	30	0.79	25	138	0.09
2	2	0.29	26	2	0.07
2	25	0.47	26	15	0.11
2	50	0.48	26	25	0.11
2	60	0.33	26	48	0.37
3	2	0.20	26	75	0.26
3	25	0.47	26	100	0.08
3	55	0.58	26	120	0.04
3	75	0.19	27	2	0.08
3	89	0.19	27	15	0.11
4	2	0.16	27	25	0.12
4	25	0.20	27	50	0.41
4	50	0.44	27	75	0.23
4	70	0.61	27	100	0.07
4	105	0.17	28	2	0.16
5	2	0.11	28	15	0.09
5	25	0.12	28	25	0.11
5	44	0.82	28	45	0.32
5	75	0.11	28	75	0.21
5	100	0.07	28	89	0.15
5	130	0.06	29	2	0.44
6	2	0.08	29	15	0.29
6	25	0.11	29	25	0.29
6	59	0.69	29	31	0.51
6	75	0.35	29	50	0.28
6	100	0.15	29	60	0.23
6	150	0.03	30	2	0.25
6	200	0.01	30	15	0.16

6	300	0.00	30	25	0.20
6	365	0.02	30	44	0.43
7	2	0.08	30	80	0.22
7	25	0.10	31	2	0.22
7	55	0.42	31	15	0.17
7	75	0.45	31	25	0.21
7	100	0.25	31	61	0.34
7	150	0.01	31	75	0.23
7	200	0.01	31	90	0.13
7	300	0.00	32	2	0.12
7	500	0.00	32	25	0.18
7	645	0.00	32	50	0.54
8	2	0.09	32	75	0.25
8	25	0.10	32	100	0.12
8	50	0.20	33	2	0.09
8	75	0.34	33	15	0.11
8	100	0.09	33	25	0.13
8	150	0.02	33	55	0.46
8	200	0.01	33	75	0.46
8	300	0.00	33	100	0.14
8	500	0.00	33	135	0.03
8	800	0.00	34	2	0.10
8	959	0.00	34	25	0.16
9	2	0.09	34	56	0.46
9	25	0.11	34	75	0.29
9	50	0.23	34	100	0.10
9	63	0.39	34	159	0.01
9	100	0.11	35	2	0.11
9	150	0.02	35	25	0.15
9	172	0.01	35	50	0.35
10	2	0.09	35	65	0.52
10	25	0.12	35	100	0.10
10	56	0.61	35	150	0.01
10	75	0.43	35	188	0.01
10	100	0.11	36	2	0.06
11	2	0.08	36	25	0.10
11	25	0.10	36	50	0.24
11	50	0.21	36	68	0.26

11	70	0.68	36	100	0.14
11	78	0.38	36	150	0.02
12	2	0.14	36	200	0.00
12	25	0.60	36	300	0.00
12	50	0.63	36	500	0.00
12	75	0.29	36	800	0.01
13	2	0.18	36	1000	0.00
13	25	0.64	36	1250	0.00
13	40	1.18	37	2	0.08
13	50	0.81	37	25	0.08
14	2	5.56	37	50	0.19
14	10	0.95	37	65	0.46
14	25	0.64	37	100	0.12
14	36	1.73	37	150	0.02
15	2	16.23	37	200	0.00
15	10	4.18	37	300	0.01
15	25	2.88	37	500	0.00
15	30	8.72	37	800	0.00
16	2	0.26	37	1000	0.00
16	10	0.26	37	1500	0.00
16	25	1.41	37	1700	0.00
16	50	0.67	38	2	0.09
17	2	0.16	38	25	0.18
17	15	0.33	38	60	0.50
17	25	1.07	38	75	0.37
17	50	0.57	38	100	0.12
17	75	0.15	38	150	0.03
18	2	0.13	38	200	0.00
18	15	0.16	38	300	0.01
18	25	0.25	38	500	0.00
18	50	0.58	38	800	0.00
18	80	0.13	38	1000	0.00
19	2	0.13	38	1500	0.00
19	15	0.12	39	2	0.19
19	25	0.23	39	25	0.19
19	46	0.45	39	50	0.37
19	75	0.23	39	65	0.56
19	100	0.13	39	100	0.06

19	115	0.07	39	150	0.02
20	2	0.15	39	200	0.00
20	25	0.17	39	300	0.00
20	50	0.32	39	500	0.01
20	60	0.54	39	800	0.00
20	75	0.25	39	1000	0.00
20	100	0.08	39	1350	0.00
20	150	0.02	40	2	0.15
20	200	0.01	40	25	0.22
20	300	0.01	40	50	0.33
20	470	0.01	40	66	0.39
21	2	0.21	40	100	0.15
21	25	0.23	40	150	0.02
21	50	0.34	40	200	0.00
21	65	0.31	40	300	0.00
21	100	0.13	40	500	0.00
21	150	0.02	40	800	0.00
21	200	0.01	40	950	0.00
21	300	0.00	41	2	0.21
21	500	0.00	41	25	0.31
21	800	0.01	41	56	0.40
21	935	0.01	41	75	0.25
22	2	0.08	41	100	0.05
22	25	0.16	41	150	0.02
22	50	0.28	42	2	0.17
22	65	0.56	42	25	0.29
22	100	0.11	42	41	0.33
22	150	0.01	42	75	0.19
22	190	0.01	42	100	0.11
23	2	0.08	42	150	0.02
23	25	0.17	43	2	0.16
23	52	0.66	43	25	0.25
23	75	0.22	43	38	0.47
23	100	0.10	43	75	0.15
23	150	0.02	43	100	0.07
23	165	0.02	43	125	0.14
24	2	0.07	44	2	0.28
24	25	0.42	44	25	0.30

24	50	0.13	44	40	0.60
24	64	0.42	44	75	0.19
24	100	0.09	44	90	0.10
24	150	0.02	45	2	0.29
25	2	0.09	45	15	0.37
25	15	0.08	45	30	0.59
25	25	0.10	45	50	0.30
25	52	0.24	45	75	0.17
