# Supplementary material for: Spatial distribution of the summer subsurface chlorophyll maximum in the North South China Sea
Source: PLoS One. 2021 Apr 7;16(4):e0248715. doi: 10.1371/journal.pone.0248715 (PMC8026054; doi:10.1371/journal.pone.0248715)
Supplement: S3 Fig — (PDF) [file pone.0248715.s003.pdf]

S3 Fig . Vertical profiles of temperature along six sections

station	Depth	Temperature	station	Depth	Temperature	station	Depth	Temperature	station	Depth	Temperature	station	Depth	Temperature
S2	1	29.1967	S44	0	28.4571	S45	1	28.436	S20	1	29.2328	S19	1	29.0684
S2	2	29.2038	S44	1	28.4846	S45	2	28.3267	S20	2	29.2341	S19	2	29.0674
S2	3	29.2044	S44	2	28.4851	S45	3	28.4391	S20	3	29.2337	S19	3	29.0643
S2	4	29.2045	S44	3	28.4648	S45	4	28.4408	S20	4	29.2322	S19	4	29.0651
S2	5	29.2043	S44	4	28.1709	S45	5	28.4408	S20	5	29.2302	S19	5	29.0651
S2	6	29.2047	S44	5	28.4789	S45	6	28.4409	S20	6	29.2318	S19	6	29.0647
S2	7	29.2039	S44	6	28.4644	S45	7	28.4391	S20	7	29.2326	S19	7	29.0697
S2	8	29.2066	S44	7	28.4608	S45	8	28.4278	S20	8	29.2401	S19	8	29.0715
S2	9	29.2065	S44	8	28.4621	S45	9	28.436	S20	9	29.2386	S19	9	29.0728
S2	10	29.201	S44	9	28.4678	S45	10	28.4344	S20	10	29.2373	S19	10	29.073
S2	11	29.1942	S44	10	28.4705	S45	11	28.4299	S20	11	29.2309	S19	11	29.0736
S2	12	29.1935	S44	11	28.4849	S45	12	27.0106	S20	12	29.2326	S19	12	29.0686
S2	13	29.1957	S44	12	28.4629	S45	13	28.4192	S20	13	29.2388	S19	13	29.0647
S2	14	29.1952	S44	13	28.4855	S45	14	28.275	S20	14	29.2386	S19	14	29.0561
S2	15	29.1959	S44	14	28.4747	S45	15	25.6835	S20	15	29.2383	S19	15	29.0677
S2	16	29.1955	S44	15	28.4707	S45	16	26.4063	S20	16	29.2357	S19	16	29.0652
S2	17	29.1978	S44	16	28.4709	S45	17	25.3067	S20	17	29.2373	S19	17	28.8613
S2	18	29.2011	S44	17	28.4388	S45	18	25.0022	S20	18	29.2366	S19	18	29.0306
S2	19	29.2492	S44	18	28.3236	S45	19	24.6435	S20	19	29.2372	S19	19	28.908
S2	20	29.2212	S44	19	28.4851	S45	20	24.5254	S20	20	29.237	S19	20	28.8325
S2	21	29.2044	S44	20	28.2717	S45	21	24.9992	S20	21	29.2425	S19	21	28.2657
S2	22	29.1662	S44	21	28.2069	S45	22	24.2202	S20	22	29.2431	S19	22	28.1426
S2	23	28.9933	S44	22	27.3098	S45	23	24.103	S20	23	29.2476	S19	23	27.8478
S2	24	28.3214	S44	23	26.9338	S45	24	23.9796	S20	24	29.2486	S19	24	27.5706
S2	25	28.1984	S44	24	26.6033	S45	25	23.6826	S20	25	29.2497	S19	25	27.3507
S2	26	27.9165	S44	25	26.4613	S45	26	24.0818	S20	26	29.2493	S19	26	27.267
S2	27	27.1505	S44	26	26.024	S45	27	23.9703	S20	27	29.242	S19	27	27.7929
S2	28	26.5118	S44	27	24.4284	S45	28	22.745	S20	28	29.1814	S19	28	27.5961
S2	29	26.7539	S44	28	24.309	S45	29	22.828	S20	29	29.2183	S19	29	27.0134
S2	30	27.4567	S44	29	24.2306	S45	30	22.3927	S20	30	29.012	S19	30	26.987
S2	31	28.1463	S44	30	24.1636	S45	31	22.199	S20	31	29.0702	S19	31	26.884
S2	32	25.043	S44	31	23.8489	S45	32	22.1453	S20	32	29.03	S19	32	27.1233
S2	33	25.7277	S44	32	23.5002	S45	33	21.8559	S20	33	28.6879	S19	33	26.659
S2	34	24.3286	S44	33	23.3919	S45	34	22.0513	S20	34	28.6231	S19	34	27.0571
S2	35	24.6333	S44	34	23.2313	S45	35	21.9551	S20	35	28.6952	S19	35	26.1727
S2	36	23.9687	S44	35	23.0426	S45	36	21.8361	S20	36	28.5903	S19	36	26.8058

S2	37	28.0504	S44	36	22.9749	S45	37	21.7281	S20	37	28.3596	S19	37	26.6924
S2	38	24.5279	S44	37	22.8732	S45	38	21.7058	S20	38	27.8288	S19	38	26.6634
S2	39	23.1494	S44	38	22.8055	S45	39	21.5769	S20	39	27.823	S19	39	25.316
S2	40	23.1949	S44	39	22.5884	S45	40	21.4819	S20	40	27.486	S19	40	25.1613
S2	41	22.805	S44	40	22.4539	S45	41	21.5961	S20	41	27.3476	S19	41	25.1386
S2	42	23.1977	S44	41	22.358	S45	42	21.5841	S20	42	27.1251	S19	42	25.1311
S2	43	22.7264	S44	42	22.1392	S45	43	21.3497	S20	43	26.8929	S19	43	25.345
S2	44	23.3992	S44	43	21.9016	S45	44	21.2765	S20	44	26.7426	S19	44	25.2169
S2	45	22.4334	S44	44	21.8114	S45	45	21.4008	S20	45	26.4491	S19	45	25.1459
S2	46	22.787	S44	45	21.7334	S45	46	20.7845	S20	46	26.2424	S19	46	25.1318
S2	47	22.1774	S44	46	21.64	S45	47	20.6526	S20	47	26.2023	S19	47	25.1194
S2	48	22.0208	S44	47	21.4616	S45	48	20.5648	S20	48	26.1969	S19	48	24.7014
S2	49	24.1881	S44	48	21.451	S45	49	20.4983	S20	49	25.9856	S19	49	24.6748
S2	50	27.5602	S44	49	20.9049	S45	50	20.5172	S20	50	25.9221	S19	50	25.0733
S2	51	21.5859	S44	50	20.6297	S45	51	20.4891	S20	51	25.8729	S19	51	24.8617
S2	52	21.4807	S44	51	20.3371	S45	52	20.3995	S20	52	25.4177	S19	52	24.5528
S2	53	21.8014	S44	52	20.2882	S45	53	20.1843	S20	53	25.3206	S19	53	24.4664
S2	54	21.4213	S44	53	20.1179	S45	54	20.1184	S20	54	25.2069	S19	54	24.6889
S2	55	21.4689	S44	54	20.0934	S45	55	20.0258	S20	55	25.1391	S19	55	24.2852
S2	56	21.4084	S44	55	20.0558	S45	56	19.8975	S20	56	24.9911	S19	56	24.6087
S2	57	21.5668	S44	56	20.0211	S45	57	19.7576	S20	57	24.6939	S19	57	24.1949
S2	58	21.4043	S44	57	19.9907	S45	58	19.5724	S20	58	24.4561	S19	58	24.5018
S2	59	21.6359	S44	58	19.797	S45	59	19.1977	S20	59	24.3496	S19	59	24.4421
S3	1	29.328	S44	59	19.5183	S45	60	19.15	S20	60	24.2731	S19	60	24.0115
S3	2	29.3391	S44	60	19.5909	S45	61	19.1282	S20	61	24.0179	S19	61	23.9417
S3	3	29.3419	S44	61	19.3651	S45	62	19.1055	S20	62	23.547	S19	62	23.6146
S3	4	29.344	S44	62	19.2829	S45	63	19.0944	S20	63	23.2378	S19	63	23.5583
S3	5	29.3378	S44	63	19.2017	S45	64	19.0723	S20	64	22.9997	S19	64	23.5303
S3	6	29.3297	S44	64	19.1764	S45	65	19.0558	S20	65	22.9877	S19	65	23.5029
S3	7	29.3008	S44	65	19.1842	S45	66	19.0482	S20	66	22.6575	S19	66	23.9339
S3	8	29.2982	S44	66	19.1639	S45	67	19.0154	S20	67	22.4616	S19	67	23.3988
S3	9	29.298	S44	67	19.1338	S45	68	18.9938	S20	68	22.3365	S19	68	23.0611
S3	10	29.2866	S44	68	19.1093	S45	69	18.9691	S20	69	22.273	S19	69	22.952
S3	11	29.2953	S44	69	19.0956	S45	70	18.9667	S20	70	22.1652	S19	70	22.6778
S3	12	29.2399	S44	70	19.1064	S45	71	18.9659	S20	71	21.9823	S19	71	22.3047
S3	13	29.2958	S44	71	19.0818	S45	72	18.9641	S20	72	21.8331	S19	72	22.1372
S3	14	29.3057	S44	72	19.0655	S45	73	18.9652	S20	73	21.7102	S19	73	23.0728
S3	15	29.3059	S44	73	19.0576	S45	74	18.9633	S20	74	21.6473	S19	74	22.813
S3	16	29.306	S44	74	19.0562	S45	75	18.9631	S20	75	21.4601	S19	75	21.8775
S3	17	29.3076	S44	75	19.0444	S45	76	18.9627	S20	76	21.2324	S19	76	22.2089
S3	18	29.3081	S44	76	19.0218	S45	77	18.9646	S20	77	21.1722	S19	77	21.9535
S3	19	29.3021	S44	77	18.956	S45	78	18.9657	S20	78	21.2317	S19	78	21.8511
S3	20	29.3211	S44	78	18.8986	S45	79	18.9638	S20	79	21.1392	S19	79	21.7436

S3	21	29.2737	S44	79	18.8849	S45	80	18.966	S20	80	21.0726	S19	80	21.8747
S3	22	29.183	S44	80	18.8776	S45	81	18.9631	S20	81	20.8778	S19	81	21.8523
S3	23	29.1267	S44	81	18.7592	S43	0	28.7354	S20	82	20.7626	S19	82	21.6319
S3	24	29.0643	S44	82	18.7006	S43	1	28.76	S20	83	20.7314	S19	83	21.4925
S3	25	29.0181	S44	83	18.7115	S43	2	28.758	S20	84	20.7097	S19	84	20.6821
S3	26	28.9884	S44	84	18.5542	S43	3	28.7601	S20	85	20.5309	S19	85	21.051
S3	27	28.4283	S44	85	18.5335	S43	4	28.7526	S20	86	20.3211	S19	86	20.7505
S3	28	26.9105	S44	86	18.5287	S43	5	28.7662	S20	87	20.0804	S19	87	20.5093
S3	29	27.7795	S44	87	18.5273	S43	6	28.7532	S20	88	19.9613	S19	88	20.3556
S3	30	26.0282	S44	88	18.5221	S43	7	28.7607	S20	89	19.9235	S19	89	20.3822
S3	31	25.3931	S44	89	18.5223	S43	8	28.7463	S20	90	19.8884	S19	90	20.3742
S3	32	25.2019	S44	90	18.5211	S43	9	28.7501	S20	91	19.8691	S19	91	20.2319
S3	33	26.6239	S44	91	18.5221	S43	10	28.7499	S20	92	19.3162	S19	92	20.2132
S3	34	24.7279	S41	1	28.6933	S43	11	28.7653	S20	93	19.2405	S19	93	20.1655
S3	35	25.847	S41	2	28.6943	S43	12	28.7653	S20	94	19.2055	S19	94	20.1176
S3	36	25.4204	S41	3	28.6873	S43	13	28.7554	S20	95	19.1854	S19	95	20.1576
S3	37	23.9559	S41	4	28.6869	S43	14	28.7422	S20	96	19.1518	S19	96	20.0418
S3	38	23.7653	S41	5	28.688	S43	15	28.5553	S20	97	19.1419	S19	97	20.0355
S3	39	24.8187	S41	6	28.6996	S43	16	28.3498	S20	98	19.092	S19	98	20.0305
S3	40	24.7401	S41	7	28.6996	S43	17	28.7323	S20	99	19.091	S19	99	20.0223
S3	41	24.1367	S41	8	28.6933	S43	18	27.1042	S20	100	19.1067	S19	100	20.0221
S3	42	23.1359	S41	9	28.6984	S43	19	26.556	S20	101	19.1029	S19	101	20.0102
S3	43	23.801	S41	10	28.6904	S43	20	26.3438	S20	102	19.0747	S19	102	19.9832
S3	44	22.7459	S41	11	28.6906	S43	21	26.2833	S20	103	19.0206	S19	103	19.9262
S3	45	23.3616	S41	12	28.6979	S43	22	26.115	S20	104	19.0425	S19	104	19.8904
S3	46	23.1115	S41	13	28.6917	S43	23	26.4576	S20	105	19.0142	S19	105	19.8091
S3	47	22.64	S41	14	28.6906	S43	24	26.3572	S20	106	18.9378	S19	106	19.754
S3	48	22.3537	S41	15	28.6925	S43	25	25.5681	S20	107	18.8594	S19	107	19.6283
S3	49	22.2565	S41	16	28.689	S43	26	25.2749	S20	108	18.8763	S19	108	19.4203
S3	50	22.1758	S41	17	28.6828	S43	27	24.7229	S20	109	18.8442	S19	109	19.2633
S3	51	22.2879	S41	18	28.6839	S43	28	24.653	S20	110	18.7635	S19	110	19.2304
S3	52	22.1092	S41	19	28.5233	S43	29	24.5074	S20	111	18.4973	S19	111	19.1608
S3	53	22.2052	S41	20	28.2822	S43	30	24.2406	S20	112	18.4644	S19	112	19.1422
S3	54	22.072	S41	21	28.6523	S43	31	23.8024	S20	113	18.3938	S19	113	19.1318
S3	55	21.925	S41	22	28.405	S43	32	23.3792	S20	114	18.2974	S19	114	19.1079
S3	56	21.8822	S41	23	27.9708	S43	33	23.65	S20	115	18.2962	S17	1	27.6864
S3	57	21.9014	S41	24	27.7795	S43	34	22.5971	S20	116	18.2822	S17	2	27.7137
S3	58	21.5843	S41	25	27.4228	S43	35	23.2864	S20	117	18.2723	S17	3	27.7035
S3	59	21.5866	S41	26	27.3563	S43	36	22.9261	S20	118	18.2674	S17	4	27.5257
S3	60	21.4738	S41	27	27.2929	S43	37	22.3819	S20	119	18.2112	S17	5	27.6173
S3	61	21.4085	S41	28	27.3928	S43	38	22.593	S20	120	18.2624	S17	6	27.4671
S3	62	21.3583	S41	29	26.8592	S43	39	22.4874	S20	121	18.1245	S17	7	27.328
S3	63	21.2947	S41	30	27.1922	S43	40	22.0721	S20	122	18.0518	S17	8	26.7619

S3	64	21.1965	S41	31	26.8163	S43	41	21.9278	S20	123	18.002	S17	9	26.7539
S3	65	21.1485	S41	32	26.6717	S43	42	22.3065	S20	124	17.7907	S17	10	26.7322
S3	66	21.1333	S41	33	26.1393	S43	43	22.2299	S20	125	17.6715	S17	11	26.4099
S3	67	21.1169	S41	34	26.2288	S43	44	21.6612	S20	126	17.6363	S17	12	26.2694
S3	68	21.1076	S41	35	25.5925	S43	45	21.4312	S20	127	17.5122	S17	13	26.0835
S3	69	21.1046	S41	36	25.2897	S43	46	21.3979	S20	128	17.3899	S17	14	25.9922
S3	70	21.105	S41	37	25.0779	S43	47	21.7771	S20	129	17.2219	S17	15	25.7903
S3	71	21.1022	S41	38	24.8381	S43	48	21.7165	S20	130	16.9054	S17	16	25.6331
S3	72	21.1007	S41	39	24.6916	S43	49	21.5197	S20	131	16.5543	S17	17	25.5504
S3	73	21.1006	S41	40	24.5079	S43	50	21.3901	S20	132	16.5372	S17	18	25.5022
S3	74	21.0967	S41	41	24.3099	S43	51	21.2432	S20	133	16.4693	S17	19	25.1854
S3	75	21.0073	S41	42	24.3304	S43	52	21.2191	S20	134	16.4107	S17	20	24.977
S3	76	21.0151	S41	43	24.0802	S43	53	21.1027	S20	135	16.4397	S17	21	24.7995
S3	77	20.9991	S41	44	23.9844	S43	54	20.9877	S20	136	16.3965	S17	22	24.692
S3	78	20.9988	S41	45	23.9261	S43	55	21.2982	S20	137	16.4226	S17	23	24.7951
S3	79	21.0024	S41	46	23.5501	S43	56	20.4554	S20	138	16.3872	S17	24	24.4257
S3	80	20.9979	S41	47	23.4878	S42	0	28.7654	S20	139	16.3754	S17	25	24.1733
S3	81	21.0007	S41	48	23.2312	S42	1	28.7671	S20	140	16.2849	S17	26	24.179
S3	82	21.0007	S41	49	23.0332	S42	2	28.7845	S20	141	16.325	S17	27	23.2525
S3	83	20.9337	S41	50	22.9314	S42	3	28.7675	S20	142	15.9521	S17	28	22.6685
S3	84	20.9234	S41	51	22.8526	S42	4	28.7847	S20	143	15.9289	S17	29	22.6792
S3	85	20.9206	S41	52	22.9031	S42	5	28.7847	S20	144	15.8098	S17	30	22.6053
S3	86	20.9019	S41	53	22.8935	S42	6	28.7838	S20	145	15.7871	S17	31	22.4666
S3	87	20.9021	S41	54	22.6222	S42	7	28.7693	S20	146	15.7505	S17	32	22.4526
S3	88	20.8982	S41	55	22.4535	S42	8	28.7847	S20	147	15.7169	S17	33	22.3024
S4	0	28.245	S41	56	22.8492	S42	9	28.768	S20	148	15.7093	S17	34	22.2999
S4	1	29.2978	S41	57	21.6685	S42	10	28.7865	S20	149	15.6905	S17	35	22.1516
S4	2	29.3004	S41	58	21.4619	S42	11	28.7862	S20	150	15.6753	S17	36	22.1472
S4	3	29.3013	S41	59	21.4228	S42	12	28.7847	S20	151	15.6333	S17	37	22.0627
S4	4	29.2884	S41	60	21.3861	S42	13	28.6978	S20	152	15.5867	S17	38	21.7823
S4	5	29.2835	S41	61	21.5391	S42	14	28.6101	S20	153	15.4716	S17	39	21.7181
S4	6	29.2881	S41	62	21.5239	S42	15	28.7576	S20	154	15.4882	S17	40	21.5931
S4	7	29.2912	S41	63	21.4986	S42	16	28.7395	S20	155	15.4571	S17	41	21.4243
S4	8	29.2803	S41	64	21.4255	S42	17	28.4459	S20	156	15.4346	S17	42	21.4308
S4	9	29.2725	S41	65	21.3449	S42	18	27.734	S20	157	15.4174	S17	43	21.0892
S4	10	29.2625	S41	66	21.2406	S42	19	27.4392	S20	158	15.3846	S17	44	20.9532
S4	11	29.2719	S41	67	20.9313	S42	20	27.3394	S20	159	15.3461	S17	45	20.6993
S4	12	29.2546	S41	68	20.8844	S42	21	26.2895	S20	160	15.3271	S17	46	20.7255
S4	13	29.2524	S41	69	21.0782	S42	22	26.8047	S20	161	15.2811	S17	47	20.6428
S4	14	29.2526	S41	70	20.6739	S42	23	25.6923	S20	162	15.2525	S17	48	20.6498
S4	15	29.2318	S41	71	20.6355	S42	24	26.0878	S20	163	15.2167	S17	49	20.5801
S4	16	29.2103	S41	72	20.5464	S42	25	25.6392	S20	164	15.1981	S17	50	20.5291
S4	17	29.2153	S41	73	20.4421	S42	26	24.3795	S20	165	15.1191	S17	51	20.5291

S4	18	29.195	S41	74	20.3814	S42	27	24.2497	S20	166	15.1055	S17	52	20.448
S4	19	29.1849	S41	75	20.3131	S42	28	24.3994	S20	167	15.0327	S17	53	20.4288
S4	20	29.1828	S41	76	20.2565	S42	29	24.0692	S20	168	15.0471	S17	54	20.4266
S4	21	29.1801	S41	77	20.2117	S42	30	24.1668	S20	169	15.0419	S17	55	20.4266
S4	22	29.1768	S41	78	20.1423	S42	31	23.9221	S20	170	14.9952	S17	56	20.3986
S4	23	29.1756	S41	79	19.8862	S42	32	23.705	S20	171	14.9407	S17	57	20.3505
S4	24	29.174	S41	80	19.8077	S42	33	23.4505	S20	172	14.883	S17	58	20.3063
S4	25	29.1737	S41	81	19.7506	S42	34	22.6668	S20	173	14.8543	S17	59	20.2757
S4	26	29.1718	S41	82	19.7053	S42	35	22.6138	S20	174	14.8475	S17	60	20.1705
S4	27	29.1693	S41	83	19.5689	S42	36	22.5481	S20	175	14.843	S17	61	20.1578
S4	28	29.1676	S41	84	19.3393	S42	37	22.3976	S20	176	14.8598	S17	62	20.0795
S4	29	29.1661	S41	85	19.0833	S42	38	22.5243	S20	177	14.8534	S17	63	19.9563
S4	30	29.1669	S41	86	19.0052	S42	39	22.4233	S20	178	14.8331	S17	64	19.9052
S4	31	29.1638	S41	87	18.9724	S42	40	22.3851	S20	179	14.8364	S17	65	19.7879
S4	32	29.1647	S41	88	18.8776	S42	41	22.3629	S20	180	14.8206	S17	66	19.7879
S4	33	29.1643	S41	89	18.9527	S42	42	22.2714	S20	181	14.8171	S17	67	19.4826
S4	34	29.1655	S41	90	18.9469	S42	43	22.2805	S20	182	14.8078	S17	68	19.4564
S4	35	29.1672	S41	91	18.7228	S42	44	21.9926	S20	183	14.7622	S17	69	19.4139
S4	36	29.1517	S41	92	18.702	S42	45	22.1686	S20	184	14.7499	S17	70	19.3948
S4	37	29.1531	S41	93	18.6309	S42	46	22.14	S20	185	14.7373	S17	71	19.3727
S4	38	29.1554	S41	94	18.5929	S42	47	22.0957	S20	186	14.7113	S17	72	19.3648
S4	39	28.9347	S41	95	18.4696	S42	48	21.6157	S20	187	14.7144	S17	73	19.345
S4	40	28.9176	S41	96	18.3938	S42	49	21.4147	S20	188	14.5857	S17	74	19.3312
S4	41	28.5424	S41	97	18.3217	S42	50	21.9276	S20	189	14.55	S18	0	27.8558
S4	42	28.4937	S41	98	18.2829	S42	51	21.7241	S20	190	14.5576	S18	1	27.9113
S4	43	28.5074	S41	99	18.231	S42	52	21.641	S20	191	14.5245	S18	2	27.9138
S4	44	28.4679	S41	100	18.2869	S42	53	21.4528	S20	172	14.883	S18	3	27.9043
S4	45	28.3786	S41	101	18.2518	S42	54	21.3437	S20	173	14.8543	S18	4	27.9008
S4	46	28.3364	S41	102	17.9773	S42	55	21.308	S20	174	14.8475	S18	5	27.9034
S4	47	28.277	S41	103	18.0628	S42	56	21.2009	S20	175	14.843	S18	6	27.9018
S4	48	28.2036	S41	104	18.0621	S42	57	21.1018	S20	176	14.8598	S18	7	27.8918
S4	49	28.0839	S41	105	18.0519	S42	58	20.9248	S20	177	14.8534	S18	8	27.8592
S4	50	27.9416	S41	106	18.0079	S42	59	20.7684	S20	178	14.8331	S18	9	27.8974
S4	51	27.8461	S41	107	17.9443	S42	60	20.6887	S20	179	14.8364	S18	10	27.8571
S4	52	27.783	S41	108	17.926	S42	61	21.0128	S20	180	14.8206	S18	11	27.8651
S4	53	27.6758	S41	109	17.5408	S42	62	20.7882	S20	181	14.8171	S18	12	27.8252
S4	54	27.4782	S41	110	17.8629	S42	63	20.7174	S20	182	14.8078	S18	13	27.8204
S4	55	27.2744	S41	111	17.3674	S42	64	20.6742	S20	183	14.7622	S18	14	27.8134
S4	56	26.8949	S41	112	17.5811	S42	65	20.438	S20	184	14.7499	S18	15	27.379
S4	57	26.8065	S41	113	17.5777	S42	66	20.1677	S20	185	14.7373	S18	16	27.6368
S4	58	26.6021	S41	114	17.5753	S42	67	20.2154	S20	186	14.7113	S18	17	27.2951
S4	59	26.3935	S41	115	17.5768	S42	68	20.1674	S20	187	14.7144	S18	18	27.2885
S4	60	26.1347	S41	116	17.2658	S42	69	20.1538	S20	188	14.5857	S18	19	27.2978

S4	61	26.0811	S41	117	17.1995	S42	70	20.1432	S20	189	14.55	S18	20	27.2936
S4	62	25.9491	S41	118	17.1335	S42	71	20.1339	S20	190	14.5576	S18	21	27.2741
S4	63	25.7655	S41	119	16.9534	S42	72	19.7685	S20	191	14.5245	S18	22	27.177
S4	64	25.4746	S41	120	17.4878	S42	73	19.7385	S22	0	29.1927	S18	23	27.1004
S4	65	24.8888	S41	121	16.8184	S42	74	19.7223	S22	1	29.129	S18	24	27.0901
S4	66	24.4581	S41	122	17.476	S42	75	19.7552	S22	2	29.1979	S18	25	27.0409
S4	67	24.201	S41	123	17.4628	S42	76	19.7418	S22	3	29.1234	S18	26	27.0188
S4	68	24.1851	S41	124	16.7311	S42	77	19.6185	S22	4	29.1392	S18	27	27.0261
S4	69	23.6896	S41	125	16.6157	S42	78	19.7148	S22	5	29.0751	S18	28	26.9923
S4	70	23.7387	S41	126	16.5388	S42	79	19.4912	S22	6	28.9427	S18	29	26.9737
S4	71	23.4377	S41	127	16.5355	S42	80	19.4465	S22	7	28.9396	S18	30	26.9544
S4	72	22.7436	S41	128	16.517	S42	81	19.4234	S22	8	29.2276	S18	31	26.9202
S4	73	22.8626	S41	129	17.0349	S42	82	19.3816	S22	9	28.8948	S18	32	26.8659
S4	74	22.3069	S41	130	16.9612	S42	83	19.2782	S22	10	28.8949	S18	33	26.8126
S4	75	22.0908	S41	131	16.4644	S42	84	19.4092	S22	11	28.8837	S18	34	26.6083
S4	76	22.1341	S41	132	16.4542	S42	85	19.1934	S22	12	28.8527	S18	35	26.533
S4	77	22.1003	S41	133	16.7626	S42	86	19.2922	S22	13	28.886	S18	36	26.3767
S4	78	21.9436	S41	134	16.6898	S42	87	19.2419	S22	14	28.8707	S18	37	26.1579
S4	79	21.5514	S41	135	16.4291	S42	88	19.194	S22	15	28.8516	S18	38	26.1249
S4	80	21.0555	S41	136	16.4144	S42	89	18.9458	S22	16	28.81	S18	39	25.9998
S4	81	20.7139	S41	137	16.3831	S42	90	19.1072	S22	17	28.733	S18	40	25.7393
S4	82	20.5517	S41	138	16.3727	S42	91	18.9319	S22	18	28.1348	S18	41	25.4297
S4	83	20.5206	S41	139	16.3676	S42	92	18.7499	S22	19	28.0841	S18	42	25.2663
S4	84	20.4288	S41	140	16.364	S42	93	18.6823	S22	20	28.0257	S18	43	25.2399
S4	85	20.2193	S41	141	16.3564	S42	94	18.6576	S22	21	27.9538	S18	44	25.1945
S4	86	20.1162	S41	142	16.3518	S42	95	18.5013	S22	22	27.9549	S18	45	25.1241
S4	87	20.0861	S41	143	16.4243	S42	96	18.4582	S22	23	27.9329	S18	46	25.1429
S4	88	20.0932	S41	144	16.4209	S42	97	18.4412	S22	24	27.9101	S18	47	25.0743
S4	89	20.0838	S41	145	16.1303	S42	98	18.374	S22	25	27.8379	S18	48	25.0597
S4	90	20.0809	S41	146	16.3816	S42	99	18.3102	S22	26	27.809	S18	49	24.6649
S4	91	20.0733	S41	147	16.3729	S42	100	18.3928	S22	27	27.8399	S18	50	24.7213
S4	92	20.0741	S41	148	16.1039	S42	101	18.0715	S22	28	27.2912	S18	51	24.6458
S4	93	20.0637	S41	149	16.334	S42	102	18.0079	S22	29	27.7635	S18	52	24.4839
S4	94	20.0633	S41	150	16.0494	S42	103	18.2323	S22	30	26.6951	S18	53	24.3131
S4	95	20.0617	S41	151	16.1444	S42	104	18.2151	S22	31	27.3004	S18	54	24.1372
S4	96	20.0649	S41	152	16.1278	S42	105	17.767	S22	32	24.9741	S18	55	24.0843
S4	97	20.0652	S41	153	16.1232	S42	106	17.7401	S22	33	24.7829	S18	56	24.0672
S4	98	20.065	S41	154	16.1158	S42	107	17.8627	S22	34	24.6757	S18	57	24.0174
S4	99	20.0626	S41	155	16.0583	S42	108	17.7558	S22	35	24.3652	S18	58	23.438
S4	100	20.0614	S40	0	28.7831	S42	109	17.7502	S22	36	25.3619	S18	59	22.9302
S4	101	20.0596	S40	1	28.8753	S42	110	17.7226	S22	37	24.9458	S18	60	22.8531
S4	102	20.0567	S40	2	28.9145	S42	111	17.7013	S22	38	24.7601	S18	61	22.8179
S4	103	20.0567	S40	3	28.9141	S42	112	17.7239	S22	39	24.5047	S18	62	22.7345

S4	104	20.0561	S40	4	28.9141	S42	113	17.7148	S22	40	23.4883	S18	63	22.6528
S4	105	20.0564	S40	5	28.8885	S42	114	17.6814	S22	41	23.2801	S18	64	22.4235
S6	1	29.3606	S40	6	28.8866	S42	115	17.6881	S22	42	23.2515	S18	65	22.3326
S6	2	29.3608	S40	7	28.9218	S42	116	17.6381	S22	43	24.1707	S18	66	22.289
S6	3	29.3472	S40	8	28.9188	S42	117	17.6213	S22	44	24.0883	S18	67	22.2241
S6	4	29.343	S40	9	28.9214	S42	118	17.377	S22	45	23.9628	S18	68	22.1495
S6	5	29.3471	S40	10	28.9224	S42	119	17.3039	S22	46	23.8269	S18	69	22.0384
S6	6	29.3658	S40	11	28.8902	S42	120	17.2833	S22	47	22.6336	S18	70	22.0042
S6	7	29.3655	S40	12	28.9225	S42	121	17.2682	S22	48	22.5922	S18	71	21.8284
S6	8	29.3457	S40	13	28.87	S42	122	17.1779	S22	49	22.423	S18	72	21.5028
S6	9	29.3341	S40	14	28.8604	S42	123	17.2894	S22	50	22.3672	S18	73	21.3979
S6	10	29.3499	S40	15	28.8421	S42	124	17.013	S22	51	23.3727	S18	74	21.2197
S6	11	29.3359	S40	16	28.9093	S42	125	16.931	S22	52	23.2939	S18	75	21.0386
S6	12	29.3177	S40	17	28.7645	S42	126	17.0542	S22	53	22.1906	S18	76	20.5234
S6	13	29.2427	S40	18	28.9045	S42	127	16.9688	S22	54	22.1412	S18	77	20.2943
S6	14	29.1215	S40	19	28.876	S42	128	16.9134	S22	55	23.0013	S18	78	20.143
S6	15	28.9909	S40	20	28.493	S42	129	16.7403	S22	56	22.8228	S18	79	20.0183
S6	16	29.2908	S40	21	28.3066	S42	130	16.7145	S22	57	21.9986	S18	80	20.017
S6	17	29.2835	S40	22	28.5034	S42	131	16.7573	S22	58	22.6776	S23	1	29.31
S6	18	29.2456	S40	23	28.3754	S42	132	16.68	S22	59	22.6609	S23	2	29.3657
S6	19	29.1553	S40	24	28.1725	S42	133	16.6652	S22	60	22.6136	S23	3	29.3799
S6	20	29.0881	S40	25	27.9465	S42	134	16.6871	S22	61	22.4938	S23	4	29.3967
S6	21	28.1451	S40	26	27.8585	S42	135	16.6826	S22	62	21.424	S23	5	29.3586
S6	22	27.9954	S40	27	27.7988	S42	136	16.5165	S22	63	22.2549	S23	6	29.3675
S6	23	27.8837	S40	28	27.6175	S42	137	16.5556	S22	64	21.7688	S23	7	29.429
S6	24	27.8046	S40	29	27.2962	S42	138	16.5315	S22	65	21.6676	S23	8	29.2153
S6	25	27.7591	S40	30	27.3516	S42	139	16.5135	S12	0	27.6224	S23	9	29.2563
S6	26	27.7243	S40	31	27.0458	S42	140	16.4922	S12	1	27.9306	S23	10	28.9974
S6	27	28.3384	S40	32	27.0134	S42	141	16.4927	S12	2	27.9194	S23	11	28.9299
S6	28	28.29	S40	33	26.5192	S42	142	16.4571	S12	3	27.7101	S23	12	28.9067
S6	29	27.3601	S40	34	26.846	S42	143	16.4576	S12	4	27.5453	S23	13	28.8938
S6	30	27.235	S40	35	26.3545	S42	144	16.4269	S12	5	27.2335	S23	14	28.8909
S6	31	28.0769	S40	36	26.1368	S42	145	16.4194	S12	6	26.8815	S23	15	28.854
S6	32	26.1534	S40	37	25.8586	S42	146	16.4082	S12	7	26.7348	S23	16	28.821
S6	33	27.964	S40	38	25.6682	S42	147	16.3895	S12	8	26.6521	S23	17	28.6937
S6	34	25.6036	S40	39	25.4157	S42	148	16.3781	S12	9	26.0459	S23	18	28.373
S6	35	24.8814	S40	40	25.6015	S42	149	16.3764	S12	10	26.4072	S23	19	28.3532
S6	36	27.8224	S40	41	25.255	S42	150	16.377	S12	11	26.0305	S23	20	28.168
S6	37	27.8004	S40	42	25.1586	S39	1	29.1553	S12	12	25.8979	S23	21	28.1754
S6	38	24.8127	S40	43	25.0512	S39	2	29.1618	S12	13	25.8231	S23	22	28.1388
S6	39	27.7607	S40	44	24.8426	S39	3	29.1643	S12	14	25.8141	S23	23	28.1153
S6	40	24.5946	S40	45	24.8908	S39	4	29.1696	S12	15	25.7981	S23	24	27.1008
S6	41	24.5006	S40	46	24.8772	S39	5	28.9213	S12	16	25.8076	S23	25	27.7126

S6	42	27.6433	S40	47	24.4008	S39	6	28.6373	S12	17	25.7906	S23	26	27.2791
S6	43	27.5937	S40	48	24.6591	S39	7	29.1688	S12	18	25.7925	S23	27	26.4444
S6	44	24.3065	S40	49	23.9169	S39	8	29.1683	S12	19	25.79	S23	28	26.6503
S6	45	27.3872	S40	50	24.2529	S39	9	29.1691	S12	20	25.8176	S23	29	26.3224
S6	46	27.3035	S40	51	24.0658	S39	10	29.1793	S12	21	25.8287	S23	30	26.2677
S6	47	24.1244	S40	52	23.526	S39	11	29.1793	S12	22	25.8195	S23	31	26.1648
S6	48	27.0027	S40	53	23.3797	S39	12	29.1801	S12	23	25.8201	S23	32	26.0869
S6	49	26.6245	S40	54	23.3655	S39	13	29.1806	S12	24	25.796	S23	33	26.0115
S6	50	26.348	S40	55	23.2733	S39	14	29.1804	S12	25	25.835	S23	34	25.4151
S6	51	23.1782	S40	56	23.3289	S39	15	29.1806	S12	26	25.7919	S23	35	25.4958
S6	52	25.9926	S40	57	22.9225	S39	16	29.1804	S12	27	25.8042	S23	36	24.8169
S6	53	22.89	S40	58	23.1078	S39	17	29.1812	S12	28	25.7933	S23	37	24.4164
S6	54	22.6723	S40	59	22.9469	S39	18	29.1803	S12	29	25.8102	S23	38	24.3569
S6	55	22.463	S40	60	22.7875	S39	19	29.179	S12	30	25.7802	S23	39	23.8151
S6	56	22.3647	S40	61	22.5746	S39	20	29.1801	S12	31	25.7883	S23	40	23.9982
S6	57	22.1965	S40	62	22.4647	S39	21	28.9935	S12	32	25.7777	S23	41	23.6313
S6	58	22.1652	S40	63	22.1761	S39	22	28.9195	S12	33	25.7649	S23	42	23.4551
S6	59	24.5548	S40	64	22.0788	S39	23	28.718	S12	34	25.755	S23	43	23.2649
S6	60	22.1284	S40	65	22.0439	S39	24	28.6451	S12	35	25.7216	S23	44	22.925
S6	61	24.4791	S40	66	22.0639	S39	25	28.55	S12	36	25.6919	S23	45	23.2037
S6	62	24.445	S40	67	21.9278	S39	26	28.5067	S12	37	25.5983	S23	46	22.8442
S6	63	24.3785	S40	68	21.8051	S39	27	28.3959	S12	38	25.6207	S23	47	22.7393
S6	64	21.9041	S40	69	21.7307	S39	28	28.213	S12	39	25.4683	S23	48	22.4279
S6	65	21.8171	S40	70	21.7044	S39	29	28.5008	S12	40	25.4836	S23	49	22.2744
S6	66	24.2373	S40	71	21.6257	S39	30	28.4548	S12	41	25.1249	S23	50	22.1948
S6	67	24.1575	S40	72	21.4454	S39	31	28.4017	S12	42	25.028	S23	51	22.2735
S6	68	21.7557	S40	73	21.3313	S39	32	25.8654	S12	43	24.8694	S23	52	22.1744
S6	69	21.7568	S40	74	21.2156	S39	33	25.7676	S12	44	24.8035	S23	53	22.0985
S6	70	21.7556	S40	75	21.0164	S39	34	26.5636	S12	45	24.5226	S23	54	21.9162
S6	71	21.7489	S40	76	20.8783	S39	35	25.9801	S12	46	24.1112	S23	55	21.795
S6	72	22.8185	S40	77	20.7167	S39	36	25.7494	S12	47	23.8252	S23	56	21.7228
S6	73	22.723	S40	78	20.3927	S39	37	24.6299	S12	48	23.7921	S23	57	21.5863
S6	74	22.6117	S40	79	20.3939	S39	38	24.4943	S12	49	23.7219	S23	58	21.5383
S6	75	21.6045	S40	80	20.3293	S39	39	24.7163	S12	50	23.2284	S23	59	21.4808
S6	76	21.5712	S40	81	20.2639	S39	40	24.4234	S12	51	22.7818	S23	60	21.4665
S6	77	22.0908	S40	82	20.2328	S39	41	23.826	S12	52	22.6716	S23	61	21.4336
S6	78	22.0416	S40	83	20.2468	S39	42	23.6599	S12	53	22.4929	S23	62	21.4315
S6	79	22.0167	S40	84	20.1567	S39	43	23.628	S12	54	22.5106	S23	63	21.3045
S6	80	21.9211	S40	85	20.0207	S39	44	23.7458	S12	55	22.3378	S23	64	21.3203
S6	81	21.7487	S40	86	19.9624	S39	45	23.6526	S12	56	22.3973	S23	65	21.2566
S6	82	21.7174	S40	87	19.9196	S39	46	23.4366	S12	57	22.3934	S23	66	21.0938
S6	83	20.8982	S40	88	19.6853	S39	47	23.4269	S12	58	22.3394	S23	67	20.8975
S6	84	21.6827	S40	89	19.5576	S39	48	23.446	S12	59	22.2103	S23	68	20.4173

S6	85	20.8097	S40	90	19.4874	S39	49	23.4388	S12	60	22.2166	S23	69	20.4056
S6	86	20.6288	S40	91	19.4429	S39	50	23.4187	S12	61	22.2477	S23	70	20.3716
S6	87	21.7116	S40	92	19.5937	S39	51	23.3722	S12	62	22.2248	S23	71	20.4053
S6	88	21.6884	S40	93	19.5086	S39	52	23.2341	S12	63	22.1849	S23	72	20.3795
S6	89	20.3571	S40	94	19.4359	S39	53	23.1044	S12	64	22.1469	S23	73	20.3006
S6	90	20.3496	S40	95	19.378	S39	54	23.0689	S12	65	22.1458	S23	74	19.9909
S6	91	20.3328	S40	96	19.3594	S39	55	22.8132	S12	66	22.1122	S23	75	20.1299
S6	92	21.5591	S40	97	19.3466	S39	56	22.7404	S12	67	22.0155	S23	76	20.0649
S6	93	21.5273	S40	98	19.2867	S39	57	22.5122	S12	68	22.0155	S23	77	19.964
S6	94	21.4649	S40	99	19.2739	S39	58	21.7821	S12	69	22.0054	S23	78	19.5649
S6	95	21.3246	S40	100	19.2305	S39	59	22.166	S12	70	21.9998	S23	79	19.5483
S6	96	20.2173	S40	101	19.2308	S39	60	21.9044	S12	71	21.9979	S23	80	19.488
S6	97	21.1062	S40	102	19.175	S39	61	21.5686	S12	72	22.003	S23	81	19.4112
S6	98	20.1541	S40	103	19.1372	S39	62	21.3956	S12	73	21.9959	S23	82	19.3757
S6	99	20.1378	S40	104	19.0647	S39	63	21.3565	S12	74	21.9905	S23	83	19.2982
S6	100	20.5286	S40	105	18.9257	S39	64	21.3386	S12	75	21.9915	S23	84	19.2415
S6	101	20.0965	S38	1	29.6077	S39	65	21.3169	S13	1	27.908	S23	85	19.1235
S6	102	20.0637	S38	2	29.6091	S39	66	21.2253	S13	2	27.8722	S23	86	19.0906
S6	103	20.3714	S38	3	29.6051	S39	67	21.1636	S13	3	27.6874	S23	87	19.4052
S6	104	19.9215	S38	4	29.5387	S39	68	21.1648	S13	4	27.6522	S23	88	19.2841
S6	105	19.8735	S38	5	29.5352	S39	69	21.1211	S13	5	27.58	S23	89	19.0313
S6	106	19.8292	S38	6	29.5408	S39	70	21.1312	S13	6	27.464	S23	90	19.0838
S6	107	20.1289	S38	7	29.5927	S39	71	21.0673	S13	7	26.605	S23	91	18.9371
S6	108	20.1327	S38	8	29.5588	S39	72	21.1032	S13	8	27.2902	S23	92	18.9293
S6	109	20.1665	S38	9	29.5153	S39	73	21.0944	S13	9	26.2043	S23	93	18.873
S6	110	19.7896	S38	10	29.519	S39	74	20.9819	S13	10	26.1806	S23	94	19.0046
S6	111	20.1498	S38	11	29.5148	S39	75	21.06	S13	11	26.1116	S23	95	18.9312
S6	112	19.6572	S38	12	29.5415	S39	76	20.9604	S13	12	26.054	S23	96	18.5974
S6	113	19.6001	S38	13	29.5408	S39	77	20.9894	S13	13	26.0364	S23	97	18.5811
S6	114	19.9179	S38	14	29.5356	S39	78	20.8317	S13	14	26.0581	S23	98	18.5575
S6	115	19.2545	S38	15	29.5131	S39	79	20.8102	S13	15	26.0505	S23	99	18.5994
S6	116	19.1426	S38	16	29.5129	S39	80	20.6149	S13	16	26.0414	S23	100	18.5147
S6	117	19.8704	S38	17	29.5129	S39	81	20.5407	S13	17	26.0253	S23	101	18.5787
S6	118	19.8631	S38	18	29.5332	S39	82	20.5229	S13	18	25.9908	S23	102	18.5396
S6	119	19.061	S38	19	29.5132	S39	83	20.5071	S13	19	25.9098	S23	103	18.4158
S6	120	19.031	S38	20	29.5227	S39	84	20.3556	S13	20	25.8129	S23	104	18.2945
S6	121	19.3972	S38	21	29.5105	S39	85	20.3145	S13	21	25.7141	S23	105	18.2373
S6	122	19.0121	S38	22	29.4243	S39	86	20.2448	S13	22	25.6619	S23	106	18.1696
S6	123	18.9588	S38	23	29.3021	S39	87	20.1669	S13	23	25.617	S23	107	18.1173
S6	124	19.0995	S38	24	28.8355	S39	88	20.0362	S13	24	25.5951	S23	108	18.086
S6	125	19.0572	S38	25	28.4125	S39	89	19.5075	S13	25	25.5691	S23	109	18.0192
S6	126	18.9821	S38	26	29.033	S39	90	19.3793	S13	26	25.5256	S23	110	18.0418
S6	127	18.9602	S38	27	27.0404	S39	91	19.7312	S13	27	25.4629	S23	111	18.0277

S6	128	18.9576	S38	28	28.403	S39	92	19.6698	S13	28	25.453	S23	112	18.0074
S6	129	18.9259	S38	29	26.3967	S39	93	19.588	S13	29	25.4396	S23	113	17.8936
S6	130	18.9104	S38	30	26.0806	S39	94	19.4778	S13	30	25.3907	S23	114	17.9713
S6	131	18.7875	S38	31	25.8967	S39	95	19.345	S13	31	25.3599	S23	115	17.7837
S6	132	18.7562	S38	32	25.7997	S39	96	18.6649	S13	32	25.3356	S23	116	17.8394
S6	133	18.7563	S38	33	25.7117	S39	97	18.4255	S13	33	25.3163	S23	117	17.7663
S6	134	18.713	S38	34	25.3984	S39	98	19.0892	S13	34	25.2902	S23	118	17.6703
S6	135	18.8015	S38	35	25.366	S39	99	18.4078	S13	35	25.2879	S23	119	17.6469
S6	136	18.7751	S38	36	25.3884	S39	100	18.849	S13	36	25.2286	S23	120	17.6707
S6	137	18.7487	S38	37	25.3221	S39	101	18.3973	S13	37	25.2121	S23	121	17.6175
S6	138	18.7164	S38	38	25.0272	S39	102	18.3816	S13	38	25.0189	S23	122	17.4069
S6	139	18.5742	S38	39	24.9966	S39	103	18.3583	S13	39	24.8017	S23	123	17.3533
S6	140	18.4192	S38	40	24.901	S39	104	18.2817	S13	40	24.7014	S23	124	17.3269
S6	141	18.2126	S38	41	24.8389	S39	105	18.2619	S13	41	24.4854	S23	125	17.2308
S6	142	18.4445	S38	42	24.7782	S39	106	18.1966	S13	42	24.2841	S23	126	17.2026
S6	143	18.3205	S38	43	24.8871	S39	107	18.1754	S13	43	23.8798	S23	127	17.1793
S6	144	17.9793	S38	44	24.6182	S39	108	18.3198	S13	44	23.723	S23	128	17.1123
S6	145	18.0149	S38	45	24.7206	S39	109	18.2468	S13	45	23.5847	S23	129	16.9775
S6	146	17.9413	S38	46	24.4716	S39	110	18.2007	S13	46	23.4384	S23	130	16.9023
S6	147	17.8887	S38	47	24.6555	S39	111	18.0829	S13	47	22.6939	S23	131	16.7091
S6	148	17.9045	S38	48	24.6319	S39	112	18.0421	S13	48	22.0132	S23	132	16.6415
S6	149	17.8134	S38	49	24.1363	S39	113	17.552	S13	49	21.9162	S23	133	16.5582
S6	150	17.577	S38	50	24.0144	S39	114	18.0017	S13	50	21.7218	S23	134	16.4824
S6	151	17.6908	S38	51	23.804	S39	115	17.9857	S15	1	29.0438	S23	135	16.4691
S6	152	17.6045	S38	52	23.9534	S39	116	17.4569	S15	2	28.9917	S23	136	16.4068
S6	153	17.5891	S38	53	23.7855	S39	117	17.4467	S15	3	28.797	S23	137	16.3407
S6	154	17.268	S38	54	23.6947	S39	118	17.737	S15	4	28.755	S23	138	16.3162
S6	155	17.2404	S38	55	23.4657	S39	119	17.4369	S15	5	28.6299	S23	139	16.2416
S6	156	17.3141	S38	56	23.4035	S39	120	17.4002	S15	6	28.4854	S23	140	16.1562
S6	157	17.1596	S38	57	23.0501	S39	121	17.3885	S15	7	28.4915	S23	141	16.0873
S6	158	16.9382	S38	58	22.971	S39	122	17.5728	S15	8	28.4676	S23	142	16.0727
S6	159	17.169	S38	59	22.6932	S39	123	17.554	S15	9	28.3446	S23	143	16.0474
S6	160	16.55	S38	60	22.6518	S39	124	17.5353	S15	10	28.3465	S23	144	15.9734
S6	161	16.6519	S38	61	22.7942	S39	125	17.2736	S15	11	28.3695	S23	145	15.9491
S6	162	16.9693	S38	62	22.4768	S39	126	17.1892	S15	12	28.3002	S23	146	15.9104
S6	163	16.4432	S38	63	22.42	S39	127	17.4413	S15	13	28.2537	S23	147	15.9045
S6	164	16.5596	S38	64	22.3031	S39	128	17.0441	S15	14	28.1579	S23	148	15.838
S6	165	16.4854	S38	65	22.0985	S39	129	17.4393	S15	15	28.1668	S23	149	15.65
S6	166	16.1123	S38	66	22.1685	S39	130	16.8736	S15	16	28.1553	S23	150	15.5454
S6	167	16.1897	S38	67	21.9836	S39	131	17.3396	S15	17	28.1505	S23	151	15.5038
S6	168	16.4102	S38	68	22.0541	S39	132	16.7895	S15	18	28.0585	S23	152	15.4478
S6	169	16.3424	S38	69	21.8047	S39	133	17.301	S15	19	28.1317	S23	153	15.4301
S6	170	16.2478	S38	70	21.7635	S39	134	17.2955	S15	20	27.8749	S23	154	15.4158

S6	171	16.2146	S38	71	21.742	S39	135	16.7088	S15	21	28.0434	S23	155	15.3946
S6	172	16.1395	S38	72	21.7258	S39	136	16.7055	S15	22	27.766	S23	156	15.3944
S6	173	15.4776	S38	73	21.5956	S39	137	17.2903	S15	23	27.4167	S23	157	15.3845
S6	174	15.7993	S38	74	21.5748	S39	138	17.2603	S15	24	27.2068	S23	158	15.3829
S6	175	15.5219	S38	75	21.4131	S39	139	17.2323	S15	25	26.6288	S23	159	15.3836
S6	176	15.5177	S38	76	21.4728	S39	140	17.1865	S15	26	25.3224	S23	160	15.3807
S6	177	15.5788	S38	77	21.398	S39	141	16.5601	S15	27	23.7659	S23	161	15.3806
S6	178	15.5667	S38	78	21.1841	S39	142	17.0902	S15	28	23.5009	S23	162	15.377
S6	179	15.5334	S38	79	21.1069	S39	143	17.0454	S15	29	23.4792	S23	163	15.3704
S6	180	15.5177	S38	80	20.9689	S39	144	17.0163	S15	30	23.4274	S23	164	15.3709
S6	181	15.3517	S38	81	20.9935	S39	145	16.3097	S16	0	27.9803	S24	1	29.1838
S6	182	15.5055	S38	82	21.2504	S39	146	16.9538	S16	1	27.7902	S24	2	29.3168
S6	183	15.3381	S38	83	20.9637	S39	147	16.8902	S16	2	27.1908	S24	3	29.326
S6	184	15.4381	S38	84	21.2011	S39	148	16.2111	S16	3	27.0652	S24	4	29.308
S6	185	15.2555	S38	85	21.1689	S39	149	16.2167	S16	4	26.8482	S24	5	29.3109
S6	186	15.3723	S38	86	20.8244	S39	150	16.2003	S16	5	26.6501	S24	6	29.2181
S6	187	15.3516	S38	87	21.0701	S39	151	16.1894	S16	6	26.6234	S24	7	29.3213
S6	188	15.341	S38	88	21.0145	S39	152	16.1841	S16	7	26.6393	S24	8	28.9361
S6	189	15.1579	S38	89	20.9593	S39	153	16.6212	S16	8	26.5921	S24	9	28.9576
S6	190	15.1395	S38	90	20.8446	S39	154	16.1508	S16	9	26.6252	S24	10	28.8531
S6	191	15.1237	S38	91	20.761	S39	155	16.5141	S16	10	26.5143	S24	11	28.8489
S6	192	15.1785	S38	92	20.3354	S39	156	16.4243	S16	11	26.5372	S24	12	28.8376
S6	193	15.1626	S38	93	20.2903	S39	157	15.9795	S16	12	26.2312	S24	13	28.8445
S6	194	15.0967	S38	94	20.1601	S39	158	16.216	S16	13	25.9502	S24	14	28.8193
S6	195	15.0964	S38	95	20.0899	S39	159	16.1614	S16	14	26.2318	S24	15	28.8292
S6	196	15.1559	S38	96	20.224	S39	160	15.9237	S16	15	24.5428	S24	16	28.8208
S6	197	15.0788	S38	97	19.967	S39	161	15.9155	S16	16	24.2815	S24	17	28.8144
S6	198	15.0263	S38	98	19.9154	S39	162	16.0317	S16	17	24.1933	S24	18	28.8137
S6	199	14.9204	S38	99	20.1082	S39	163	15.9989	S16	18	24.0722	S24	19	28.8131
S6	200	15.0957	S38	100	20.0706	S39	164	15.9743	S16	19	24.4893	S24	20	28.8056
S7	1	29.0965	S38	101	19.7399	S39	165	15.9069	S16	20	23.4604	S24	21	28.7221
S7	2	29.0906	S38	102	19.9314	S39	166	15.8739	S16	21	23.2708	S24	22	28.7603
S7	3	29.0939	S38	103	19.2889	S39	167	15.8514	S16	22	24.1395	S24	23	28.6431
S7	4	29.0899	S38	104	19.2066	S39	168	15.7259	S16	23	24.0377	S24	24	28.6897
S7	5	29.0693	S38	105	19.1197	S39	169	15.7858	S16	24	23.7931	S24	25	28.594
S7	6	29.0711	S38	106	19.0929	S39	170	15.7442	S16	25	23.3095	S24	26	28.1577
S7	7	29.0702	S38	107	19.0607	S39	171	15.7168	S16	26	21.91	S24	27	28.6196
S7	8	29.0899	S38	108	19.0073	S39	172	15.6311	S16	27	22.3749	S24	28	27.2767
S7	9	29.0879	S38	109	19.2201	S39	173	15.5922	S16	28	22.0811	S24	29	27.1194
S7	10	29.0577	S38	110	18.9076	S39	174	15.4992	S16	29	21.8451	S24	30	26.9101
S7	11	29.0742	S38	111	19.1251	S39	175	15.4858	S16	30	21.8019	S24	31	28.0217
S7	12	29.0673	S38	112	19.0412	S39	176	15.4539	S16	31	21.7468	S24	32	26.7182
S7	13	29.0597	S38	113	18.9369	S39	177	15.5531	S16	32	21.6489	S24	33	27.2936

S7	14	29.0249	S38	114	18.9048	S39	178	15.2841	S16	33	21.6645	S24	34	27.2279
S7	15	29.0384	S38	115	18.3486	S39	179	15.2547	S16	34	21.6497	S24	35	27.0312
S7	16	28.9928	S38	116	18.8661	S39	180	15.2334	S16	35	21.6236	S24	36	26.8536
S7	17	28.9957	S38	117	18.2464	S39	181	15.0987	S16	36	21.603	S24	37	25.9104
S7	18	28.9906	S38	118	18.6248	S39	182	14.9912	S16	37	21.6028	S24	38	25.0972
S7	19	28.9593	S38	119	18.4586	S39	183	14.9484	S16	38	21.6044	S24	39	25.2745
S7	20	28.9067	S38	120	18.3136	S39	184	14.9255	S16	39	21.6015	S24	40	25.1328
S7	21	28.9367	S38	121	18.2587	S39	185	15.3682	S16	40	21.6018	S24	41	25.006
S7	22	28.7145	S38	122	18.2201	S39	186	15.3376	S16	41	21.6028	S24	42	24.8213
S7	23	28.6308	S38	123	17.8139	S39	187	15.2236	S16	42	21.6024	S24	43	25.7459
S7	24	28.751	S38	124	18.0991	S39	188	15.0395	S16	43	21.6016	S24	44	25.3145
S7	25	28.6314	S38	125	17.9696	S39	189	14.9795	S16	44	21.6016	S24	45	25.1606
S7	26	27.7148	S38	126	17.7198	S39	190	14.5843	S16	45	21.6018	S24	46	25.0893
S7	27	28.276	S38	127	17.7095	S39	191	14.9228	S16	46	21.6012	S24	47	24.8005
S7	28	28.0186	S38	128	17.6916	S39	192	14.8001	S16	47	21.5978	S24	48	24.4977
S7	29	27.445	S38	129	17.6788	S39	193	14.4529	S16	48	21.6001	S24	49	23.2025
S7	30	27.9383	S38	130	17.6718	S39	194	14.69	S16	49	21.5998	S24	50	24.339
S7	31	26.4159	S38	131	17.7133	S39	195	14.3647	S16	50	21.5998	S24	51	22.4949
S7	32	27.715	S38	132	17.7042	S39	196	14.3057	S16	51	21.5987	S24	52	24.2422
S7	33	27.5673	S38	133	17.7006	S39	197	14.272	S16	52	21.5968	S24	53	22.1193
S7	34	26.1956	S38	134	17.6084	S39	198	14.2357	S30	1	29.269	S24	54	22.0697
S7	35	26.1148	S38	135	17.5327	S39	199	14.188	S30	2	29.2642	S24	55	23.535
S7	36	26.6842	S38	136	17.598	S39	200	14.164	S30	3	29.2918	S24	56	21.9246
S7	37	25.8431	S38	137	17.5324	S37	1	29.5986	S30	4	29.2541	S24	57	23.1421
S7	38	26.4625	S38	138	17.388	S37	2	29.6075	S30	5	29.1632	S24	58	21.3737
S7	39	26.3938	S38	139	17.4181	S37	3	29.5353	S30	6	29.0697	S24	59	22.8284
S7	40	26.2984	S38	140	17.3739	S37	4	29.5338	S30	7	28.9666	S21	1	29.1782
S7	41	25.0511	S38	141	17.2762	S37	5	29.6031	S30	8	28.8637	S21	2	29.1782
S7	42	26.1462	S38	142	17.2048	S37	6	29.5356	S30	9	28.8259	S21	3	29.1782
S7	43	24.7807	S38	143	17.079	S37	7	29.6035	S30	10	28.5647	S21	4	29.1756
S7	44	25.9985	S38	144	16.9844	S37	8	29.5391	S30	11	28.5927	S21	5	29.1775
S7	45	24.5984	S38	145	16.9428	S37	9	29.6034	S30	12	28.6374	S21	6	29.1777
S7	46	25.6791	S38	146	16.8054	S37	10	29.5409	S30	13	28.6249	S21	7	29.1828
S7	47	25.6547	S38	147	16.7048	S37	11	29.6042	S30	14	28.6647	S21	8	29.1826
S7	48	24.2547	S38	148	16.6318	S37	12	29.6047	S30	15	28.1109	S21	9	29.1742
S7	49	24.2586	S38	149	16.4962	S37	13	29.6066	S30	16	27.9523	S21	10	29.1801
S7	50	24.8895	S38	150	16.4027	S37	14	29.5411	S30	17	28.5988	S21	11	29.1812
S7	51	24.1696	S38	151	16.2941	S37	15	29.6023	S30	18	27.4172	S21	12	29.1821
S7	52	24.6424	S38	152	16.2625	S37	16	29.5411	S30	19	28.4896	S21	13	29.1815
S7	53	24.5226	S38	153	16.2527	S37	17	29.5996	S30	20	26.8364	S21	14	29.1823
S7	54	23.9138	S38	154	16.2455	S37	18	29.5389	S30	21	28.2127	S21	15	29.1808
S7	55	23.8419	S38	155	16.2276	S37	19	29.6021	S30	22	28.0877	S21	16	29.1806
S7	56	24.2706	S38	156	16.1012	S37	20	29.5387	S30	23	25.1544	S21	17	29.1806

S7	57	24.1741	S36	1	29.2184	S37	21	29.5387	S30	24	24.9746	S21	18	29.1797
S7	58	23.5593	S36	2	29.2708	S37	22	29.5955	S30	25	27.3323	S21	19	29.1766
S7	59	23.526	S36	3	29.3402	S37	23	29.5798	S30	26	24.7852	S21	20	29.1788
S7	60	23.5125	S36	4	29.0416	S37	24	29.5389	S30	27	24.5231	S21	21	29.1791
S7	61	23.6557	S36	5	29.2215	S37	25	29.5391	S30	28	26.4513	S21	22	29.1689
S7	62	23.6203	S36	6	29.3008	S37	26	29.5374	S30	29	25.9597	S21	23	29.176
S7	63	23.3785	S36	7	29.2883	S37	27	29.3413	S30	30	24.1008	S21	24	29.1186
S7	64	23.5429	S36	8	29.2717	S37	28	29.2333	S30	31	23.984	S21	25	29.0956
S7	65	23.2769	S36	9	29.2032	S37	29	29.5278	S30	32	24.851	S21	26	28.992
S7	66	23.0953	S36	10	29.1878	S37	30	29.5168	S30	33	23.6382	S21	27	28.8771
S7	67	23.3468	S36	11	29.1405	S37	31	28.4637	S30	34	23.4021	S21	28	28.6101
S7	68	22.6058	S36	12	29.084	S37	32	29.4515	S30	35	23.1338	S21	29	28.1284
S7	69	22.5492	S36	13	28.9303	S37	33	29.398	S30	36	23.9447	S21	30	27.4297
S7	70	22.7997	S36	14	28.8764	S37	34	27.7568	S30	37	22.675	S21	31	27.7151
S7	71	22.3701	S36	15	28.9129	S37	35	27.3841	S30	38	22.5948	S21	32	26.7612
S7	72	22.3457	S36	16	28.8547	S37	36	28.9224	S30	39	22.2781	S21	33	27.0381
S7	73	22.2862	S36	17	28.8281	S37	37	27.2904	S30	40	22.939	S21	34	26.0343
S7	74	22.5054	S36	18	28.8126	S37	38	28.6372	S30	41	21.8212	S21	35	25.9644
S7	75	21.989	S36	19	28.8219	S37	39	28.5857	S30	42	22.6363	S21	36	25.9572
S7	76	22.3811	S36	20	28.8206	S37	40	28.3979	S30	43	22.4047	S21	37	25.9176
S7	77	22.3266	S36	21	28.6632	S37	41	27.002	S30	44	22.2442	S21	38	25.896
S7	78	21.5512	S36	22	28.8074	S37	42	27.9005	S30	45	21.2032	S21	39	25.8514
S7	79	22.205	S36	23	28.6064	S37	43	27.8695	S30	46	21.7255	S21	40	25.7809
S7	80	21.2307	S36	24	28.6149	S37	44	25.764	S30	47	20.8703	S21	41	25.3613
S7	81	21.1846	S36	25	28.4089	S37	45	27.2194	S30	48	21.3227	S21	42	25.1914
S7	82	21.1319	S36	26	28.3794	S37	46	24.9594	S30	49	21.1762	S21	43	25.1552
S7	83	21.6947	S36	27	28.4868	S37	47	26.9446	S30	50	20.9782	S21	44	25.0545
S7	84	21.6257	S36	28	28.3267	S37	48	26.9271	S30	51	20.6706	S21	45	24.7556
S7	85	20.8474	S36	29	28.2404	S37	49	23.9577	S30	52	20.5872	S21	46	24.882
S7	86	20.7589	S36	30	28.1976	S37	50	23.5689	S30	53	20.5237	S21	47	24.6866
S7	87	20.5948	S36	31	28.1551	S37	51	25.8306	S30	54	20.6742	S21	48	24.6164
S7	88	21.5943	S36	32	27.8398	S37	52	25.6757	S30	55	20.3742	S21	49	24.438
S7	89	20.1193	S36	33	26.9669	S37	53	25.3744	S30	56	20.5678	S21	50	23.9134
S7	90	21.3068	S36	34	26.5767	S37	54	24.9395	S30	57	20.1374	S21	51	23.6597
S7	91	21.2593	S36	35	26.2074	S37	55	23.5237	S30	58	20.3559	S21	52	23.4766
S7	92	21.2037	S36	36	26.1785	S37	56	23.51	S30	59	20.2222	S21	53	23.3841
S7	93	21.1146	S36	37	26.0808	S37	57	23.4643	S30	60	19.9578	S21	54	23.3126
S7	94	19.3878	S36	38	26.1552	S37	58	23.4436	S30	61	20.0452	S21	55	23.1113
S7	95	21.0254	S36	39	25.4798	S37	59	23.7641	S30	62	19.9748	S21	56	22.7011
S7	96	20.882	S36	40	25.5794	S37	60	23.4974	S30	63	19.833	S21	57	22.395
S7	97	19.3161	S36	41	25.3104	S37	61	22.9805	S30	64	19.7721	S21	58	21.9924
S7	98	20.5696	S36	42	25.2008	S37	62	23.2976	S30	65	19.4905	S21	59	22.0619
S7	99	20.5203	S36	43	25.1178	S37	63	23.2763	S30	66	19.6088	S21	60	21.8229

S7	100	20.4124	S36	44	24.5743	S37	64	22.9015	S30	67	19.3677	S21	61	21.6341
S7	101	20.22	S36	45	24.4325	S37	65	23.2986	S30	68	19.4107	S21	62	21.8462
S7	102	19.1187	S36	46	23.9803	S37	66	23.3073	S30	69	19.2174	S21	63	21.804
S7	103	18.9973	S36	47	23.9558	S37	67	23.2511	S30	70	19.1792	S21	64	21.3185
S7	104	19.7874	S36	48	23.6565	S37	68	22.4078	S30	71	19.1955	S21	65	21.2314
S7	105	19.7369	S36	49	23.4238	S37	69	23.1297	S30	72	19.1613	S21	66	21.1219
S7	106	18.95	S36	50	23.202	S37	70	23.0797	S30	73	19.1258	S21	67	21.08
S7	107	18.8883	S36	51	23.1885	S37	71	22.0794	S30	74	19.1005	S21	68	21.0887
S7	108	18.8632	S36	52	23.1942	S37	72	21.9663	S30	75	19.0988	S21	69	20.8752
S7	109	19.4721	S36	53	23.3786	S37	73	21.9127	S30	76	19.0941	S21	70	20.9143
S7	110	18.8051	S36	54	23.3693	S37	74	22.6932	S30	77	19.0906	S21	71	20.5251
S7	111	18.7376	S36	55	23.3569	S37	75	22.6697	S30	78	19.0918	S21	72	20.4879
S7	112	18.7263	S36	56	23.3572	S37	76	21.3426	S30	79	19.0892	S21	73	20.5589
S7	113	19.2219	S36	57	22.9787	S37	77	21.3171	S30	80	19.0917	S21	74	20.3343
S7	114	19.1973	S36	58	22.9707	S37	78	21.2929	S10	1	28.9781	S21	75	20.2846
S7	115	18.5887	S36	59	22.9574	S37	79	21.2365	S10	2	28.9864	S21	76	20.083
S7	116	19.1003	S36	60	23.3355	S37	80	21.8508	S10	3	28.9794	S21	77	19.9898
S7	117	19.0911	S36	61	22.8067	S37	81	21.0994	S10	4	28.9891	S21	78	19.9554
S7	118	18.4448	S36	62	23.2841	S37	82	21.6592	S10	5	28.9891	S21	79	19.8443
S7	119	18.4017	S36	63	23.2323	S37	83	21.6247	S10	6	28.9757	S21	80	19.8443
S7	120	18.3552	S36	64	23.1414	S37	84	21.605	S10	7	28.9909	S21	81	19.7969
S7	121	18.3402	S36	65	22.2355	S37	85	20.843	S10	8	28.9737	S21	82	19.7836
S7	122	18.3131	S36	66	22.1957	S37	86	20.7733	S10	9	28.9909	S21	83	19.6446
S7	123	18.3014	S36	67	22.6898	S37	87	21.2293	S10	10	28.9733	S21	84	19.6227
S7	124	18.7708	S36	68	22.3884	S37	88	20.6039	S10	11	28.9706	S21	85	19.5692
S7	125	18.7616	S36	69	22.0691	S37	89	20.2706	S10	12	28.9704	S21	86	19.506
S7	126	18.7534	S36	70	22.3569	S37	90	20.1933	S10	13	28.9534	S21	87	19.4106
S7	127	18.0631	S36	71	22.3421	S37	91	20.1221	S10	14	28.933	S21	88	19.3826
S7	128	18.6503	S36	72	22.3365	S37	92	19.9892	S10	15	28.9239	S21	89	19.348
S7	129	18.5678	S36	73	21.6431	S37	93	20.8757	S10	16	28.903	S21	90	19.3073
S7	130	17.9343	S36	74	22.1455	S37	94	19.8452	S10	17	28.9363	S21	91	19.2191
S7	131	17.9268	S36	75	22.1195	S37	95	19.7749	S10	18	28.9069	S21	92	19.1963
S7	132	17.9234	S36	76	22.094	S37	96	19.5835	S10	19	28.8621	S21	93	19.1781
S7	133	17.9189	S36	77	22.0693	S37	97	20.1574	S10	20	28.8959	S21	94	19.1419
S7	134	18.436	S36	78	20.798	S37	98	20.1489	S10	21	28.8057	S21	95	19.1014
S7	135	18.3795	S36	79	20.6837	S37	99	20.1242	S10	22	28.7515	S21	96	19.0588
S7	136	18.3519	S36	80	21.7873	S37	100	20.0607	S10	23	28.7233	S21	97	18.9627
S7	137	18.1015	S36	81	21.523	S37	101	20.056	S10	24	28.8103	S21	98	18.9538
S7	138	18.0318	S36	82	20.3291	S37	102	19.1878	S10	25	28.6438	S21	99	18.9479
S7	139	17.4235	S36	83	20.1719	S37	103	19.1315	S10	26	28.6196	S21	100	18.9333
S7	140	17.2822	S36	84	21.2348	S37	104	18.9453	S10	27	28.6219	S21	101	18.9304
S7	141	17.2605	S36	85	21.1282	S37	105	19.9024	S10	28	28.5951	S21	102	18.7551
S7	142	18.0017	S36	86	20.9861	S37	106	18.8203	S10	29	28.5169	S21	103	18.6997

S7	143	17.9959	S36	87	19.9689	S37	107	19.7969	S10	30	28.5641	S21	104	18.812
S7	144	17.9928	S36	88	19.9503	S37	108	18.7751	S10	31	28.3985	S21	105	18.3816
S7	145	17.0327	S36	89	19.8466	S37	109	19.632	S10	32	28.371	S21	106	18.7696
S7	146	17.8179	S36	90	20.7134	S37	110	19.6019	S10	33	28.3304	S21	107	18.6179
S7	147	17.0135	S36	91	19.4854	S37	111	19.5876	S10	34	28.2816	S21	108	18.1373
S7	148	16.9532	S36	92	20.3887	S37	112	19.5547	S10	35	28.3412	S21	109	18.0941
S7	149	16.8276	S36	93	20.3145	S37	113	18.572	S10	36	28.2959	S21	110	18.2657
S7	150	17.2442	S36	94	20.2672	S37	114	18.568	S10	37	27.9291	S21	111	18.0215
S7	151	16.7662	S36	95	20.1439	S37	115	18.5663	S10	38	28.1279	S21	112	18.1017
S7	152	17.089	S36	96	19.9512	S37	116	19.3717	S10	39	27.8483	S21	113	18.0573
S7	153	17.0836	S36	97	18.8331	S37	117	19.2315	S10	40	27.7788	S21	114	18.0468
S7	154	17.0709	S36	98	18.7802	S37	118	19.1403	S10	41	27.5999	S21	115	17.9764
S7	155	17.0577	S36	99	19.278	S37	119	18.5318	S10	42	27.8733	S21	116	17.9588
S7	156	16.988	S36	100	19.1957	S37	120	19.0317	S10	43	27.7663	S21	117	17.7768
S7	157	16.8542	S36	101	19.1325	S37	121	18.4941	S10	44	27.5051	S21	118	17.8841
S7	158	16.7418	S36	102	18.4092	S37	122	18.4853	S10	45	27.5034	S21	119	17.7918
S7	159	16.4198	S36	103	18.6693	S37	123	18.4393	S10	46	26.9765	S21	120	17.6571
S7	160	16.3957	S36	104	18.5954	S37	124	18.4166	S10	47	27.4874	S21	121	17.5912
S7	161	16.5481	S36	105	18.5458	S37	125	18.7035	S10	48	26.7585	S21	122	17.4643
S7	162	16.4819	S36	106	18.1327	S37	126	18.3229	S10	49	27.2641	S21	123	17.4545
S7	163	16.4368	S36	107	18.4094	S37	127	18.6638	S10	50	26.5551	S21	124	17.3671
S7	164	16.3979	S36	108	17.843	S37	128	18.6319	S10	51	25.9271	S21	125	17.3362
S7	165	15.9296	S36	109	17.7926	S37	129	18.6259	S10	52	26.6044	S21	126	17.3656
S7	166	15.9181	S36	110	17.7052	S37	130	18.0105	S10	53	25.8244	S21	127	17.3337
S7	167	16.1676	S36	111	18.2292	S37	131	18.6196	S10	54	25.4591	S21	128	17.2392
S7	168	16.0681	S36	112	18.1066	S37	132	18.6164	S10	55	25.6306	S21	129	17.2252
S7	169	16.0443	S36	113	17.6418	S37	133	17.826	S10	56	25.5374	S21	130	17.1942
S7	170	15.9621	S36	114	17.8493	S37	134	18.6008	S10	57	25.529	S21	131	17.1837
S7	171	15.9259	S36	115	17.885	S37	135	18.5972	S10	58	25.2108	S21	132	17.0555
S7	172	15.7254	S36	116	17.8098	S37	136	18.593	S10	59	25.3111	S21	133	16.9847
S7	173	15.8665	S36	117	17.8016	S37	137	17.6417	S10	60	25.2764	S21	134	16.8729
S7	174	15.8508	S36	118	17.4778	S37	138	17.5491	S10	61	25.0618	S21	135	16.8406
S7	175	15.6615	S36	119	17.4477	S37	139	17.286	S10	62	25.0983	S21	136	16.8053
S7	176	15.601	S36	120	17.4057	S37	140	17.2373	S10	63	24.9076	S29	0	28.6385
S7	177	15.6206	S36	121	17.3955	S35	0	29.5898	S10	64	24.9207	S29	1	28.973
S7	178	15.7027	S36	122	17.5743	S35	1	29.5979	S10	65	24.7972	S29	2	28.9095
S7	179	15.6909	S36	123	17.3318	S35	2	29.6138	S10	66	24.7129	S29	3	28.6189
S7	180	15.6743	S36	124	17.3126	S35	3	29.6125	S10	67	24.6035	S29	4	28.5821
S7	181	15.3821	S36	125	17.2108	S35	4	29.588	S10	68	24.5967	S29	5	28.0625
S7	182	15.2921	S36	126	17.2021	S35	5	29.4807	S10	69	24.6106	S29	6	28.1972
S7	183	15.6233	S36	127	17.1874	S35	6	29.4078	S10	70	24.5537	S29	7	28.1833
S7	184	15.5948	S36	128	17.1677	S35	7	29.5201	S10	71	23.3138	S29	8	28.1314
S7	185	15.5459	S36	129	17.0662	S35	8	29.1423	S10	72	23.6716	S29	9	28.1715

S7	186	15.1738	S36	130	17.026	S35	9	29.1193	S10	73	23.5625	S29	10	28.1548
S7	187	15.3187	S36	131	17.0069	S35	10	29.1729	S10	74	23.1924	S29	11	28.1458
S7	188	15.1003	S36	132	16.9946	S35	11	29.0987	S10	75	23.219	S29	12	28.1395
S7	189	15.2662	S36	133	16.7017	S35	12	29.1254	S10	76	23.1487	S29	13	28.126
S7	190	14.9844	S36	134	16.9047	S35	13	29.1141	S10	77	22.5017	S29	14	28.0155
S7	191	15.2293	S36	135	16.5938	S35	14	28.8114	S10	78	22.8096	S29	15	27.9793
S7	192	14.907	S36	136	16.5502	S35	15	28.4665	S10	79	22.3846	S29	16	28.0302
S7	193	15.1313	S36	137	16.5116	S35	16	29.0897	S10	80	22.2299	S29	17	28.0059
S7	194	14.9738	S36	138	16.2759	S35	17	29.0245	S10	81	21.7803	S29	18	27.248
S7	195	14.8127	S36	139	16.2116	S35	18	28.2889	S10	82	21.5401	S29	19	26.5632
S7	196	14.7851	S36	140	16.4018	S35	19	28.1567	S10	83	21.9438	S29	20	26.3271
S7	197	14.7364	S36	141	16.1283	S35	20	28.4184	S10	84	21.1238	S29	21	26.5887
S7	198	14.8679	S36	142	16.2822	S35	21	28.3789	S10	85	21.3956	S29	22	26.3622
S7	199	14.6538	S36	143	16.1584	S35	22	28.3176	S10	86	21.127	S29	23	26.1619
S7	200	14.6363	S36	144	15.9899	S35	23	28.2827	S10	87	21.0335	S29	24	25.0877
S8	1	29.6199	S36	145	15.8458	S35	24	27.7103	S10	88	20.2719	S29	25	25.961
S8	2	29.7106	S36	146	15.7701	S35	25	28.0512	S10	89	20.0053	S29	26	24.8216
S8	3	29.6214	S36	147	15.9179	S35	26	27.4589	S10	90	20.057	S29	27	24.4234
S8	4	29.6206	S36	148	15.7166	S35	27	27.8244	S10	91	19.9363	S29	28	24.929
S8	5	29.6182	S36	149	15.7599	S35	28	27.0674	S10	92	19.8437	S29	29	24.6753
S8	6	29.6274	S36	150	15.7525	S35	29	27.742	S10	93	19.8283	S29	30	23.383
S8	7	29.7081	S36	151	15.7274	S35	30	27.5676	S10	94	19.8137	S29	31	23.9422
S8	8	29.5912	S36	152	15.6393	S35	31	26.113	S10	95	19.8226	S29	32	23.5219
S8	9	29.6754	S36	153	15.5329	S35	32	27.0694	S10	96	19.7891	S29	33	23.2894
S8	10	29.5889	S36	154	15.4235	S35	33	26.7525	S10	97	19.7324	S29	34	23.2303
S8	11	29.4999	S36	155	15.2961	S35	34	25.6227	S10	98	19.7194	S29	35	22.3429
S8	12	29.3859	S36	156	15.4076	S35	35	26.2399	S10	99	19.7161	S29	36	21.8858
S8	13	29.3652	S36	157	15.3322	S35	36	25.4178	S10	100	19.7099	S29	37	22.8349
S8	14	29.3399	S36	158	15.1913	S35	37	26.0603	S11	1	28.7402	S29	38	21.9537
S8	15	29.3551	S36	159	15.1626	S35	38	25.0543	S11	2	28.7494	S29	39	21.8696
S8	16	29.3401	S36	160	15.1418	S35	39	24.7854	S11	3	28.7173	S29	40	22.5548
S8	17	29.3284	S36	161	14.9092	S35	40	25.7052	S11	4	28.7393	S29	41	21.1913
S8	18	29.3238	S36	162	14.797	S35	41	24.0783	S11	5	28.7404	S29	42	22.2964
S8	19	29.3371	S36	163	14.7849	S35	42	25.3708	S11	6	28.7564	S29	43	22.0818
S8	20	29.337	S36	164	14.7458	S35	43	25.1368	S11	7	28.7484	S29	44	21.2204
S8	21	29.3363	S36	165	14.861	S35	44	25.0334	S11	8	28.7487	S29	45	21.9174
S8	22	29.2635	S36	166	14.7574	S35	45	24.6671	S11	9	28.7497	S29	46	20.5022
S8	23	29.2561	S36	167	14.5563	S35	46	23.1844	S11	10	28.6866	S29	47	21.6849
S8	24	29.3308	S36	168	14.4625	S35	47	23.068	S11	11	28.6276	S29	48	20.3231
S8	25	29.3225	S36	169	14.5942	S35	48	24.0549	S11	12	28.5848	S29	49	20.215
S8	26	29.242	S36	170	14.592	S35	49	23.016	S11	13	28.4859	S29	50	19.8594
S8	27	29.2208	S36	171	14.5876	S35	50	23.0024	S11	14	28.2771	S29	51	19.8323
S8	28	29.2807	S36	172	14.5624	S35	51	23.6844	S11	15	28.2341	S29	52	19.8488

S8	29	29.2618	S36	173	14.2485	S35	52	23.4581	S11	16	28.222	S29	53	19.8676
S8	30	29.2348	S36	174	14.5249	S35	53	23.2548	S11	17	28.2261	S29	54	19.8728
S8	31	29.2199	S36	175	14.5074	S35	54	22.8691	S11	18	28.2106	S29	55	19.8311
S8	32	29.2161	S36	176	14.2325	S35	55	22.7638	S11	19	28.2045	S29	56	19.8808
S8	33	29.2271	S36	177	14.4418	S35	56	22.5467	S11	20	28.2007	S29	57	19.732
S8	34	29.2096	S36	178	14.4288	S35	57	22.3238	S11	21	28.1941	S29	58	19.7747
S8	35	29.132	S36	179	14.3971	S35	58	23.0302	S11	22	28.193	S29	59	19.647
S8	36	28.5303	S36	180	14.3506	S35	59	23.0068	S11	23	28.1906	S25	0	29.2495
S8	37	28.4201	S36	181	14.1502	S35	60	22.9616	S11	24	28.1851	S25	1	28.9255
S8	38	28.2897	S36	182	14.1364	S35	61	21.8487	S11	25	28.1489	S25	2	28.8259
S8	39	28.0417	S36	183	14.1282	S35	62	22.5879	S11	26	28.1494	S25	3	29.3148
S8	40	27.8481	S36	184	14.2962	S35	63	22.2461	S11	27	28.1304	S25	4	29.2959
S8	41	27.6355	S36	185	14.2865	S35	64	21.5364	S11	28	28.0941	S25	5	29.2423
S8	42	27.7915	S36	186	14.1943	S35	65	21.8694	S11	29	28.0694	S25	6	29.1972
S8	43	27.0252	S36	187	14.1033	S35	66	21.7934	S11	30	28.0421	S25	7	29.1498
S8	44	27.4881	S36	188	14.0156	S35	67	21.0227	S11	31	28.0448	S25	8	28.8926
S8	45	27.4316	S36	189	14.01	S35	68	20.8135	S11	32	27.994	S25	9	28.832
S8	46	27.1196	S36	190	14.0396	S35	69	20.6998	S11	33	27.9947	S25	10	28.892
S8	47	26.8686	S36	191	14.0279	S35	70	20.636	S11	34	28.0211	S25	11	28.8098
S8	48	26.7144	S36	192	13.9056	S35	71	20.9528	S11	35	28.0104	S25	12	28.8019
S8	49	26.1228	S36	193	13.8506	S35	72	20.3678	S11	36	27.9532	S25	13	28.8127
S8	50	26.1484	S36	194	13.9743	S35	73	20.2763	S11	37	27.9036	S25	14	28.736
S8	51	26.0678	S36	195	13.9714	S35	74	20.4372	S11	38	27.8998	S25	15	28.6921
S8	52	25.8728	S36	196	13.9482	S35	75	20.3462	S11	39	27.9657	S25	16	28.5804
S8	53	24.8574	S36	197	13.9169	S35	76	20.2092	S11	40	27.9558	S25	17	28.7167
S8	54	25.2995	S36	198	13.9004	S35	77	20.0053	S11	41	27.9443	S25	18	27.9662
S8	55	24.5383	S36	199	13.9009	S35	78	20.0015	S11	42	27.9357	S25	19	27.1799
S8	56	24.8462	S36	200	13.5155	S35	79	19.8009	S11	43	27.7842	S25	20	27.0405
S8	57	24.6642	S33	1	29.4735	S35	80	19.6248	S11	44	27.8392	S25	21	27.4645
S8	58	24.4239	S33	2	29.4862	S35	81	19.8422	S11	45	27.6765	S25	22	27.0884
S8	59	23.9499	S33	3	29.3734	S35	82	19.4601	S11	46	27.6424	S25	23	26.9961
S8	60	23.7411	S33	4	29.4561	S35	83	19.393	S11	47	27.678	S25	24	26.9408
S8	61	24.2527	S33	5	29.5002	S35	84	19.4891	S11	48	27.6348	S25	25	26.4993
S8	62	24.1988	S33	6	29.5234	S35	85	19.3935	S11	49	27.4742	S25	26	26.7726
S8	63	23.061	S33	7	29.1591	S35	86	19.154	S11	50	27.3064	S25	27	26.6815
S8	64	23.8895	S33	8	29.215	S35	87	19.1628	S11	51	27.325	S25	28	26.3527
S8	65	23.741	S33	9	29.1502	S35	88	19.0723	S11	52	27.2312	S25	29	26.4405
S8	66	22.895	S33	10	29.0684	S35	89	19.055	S11	53	27.263	S25	30	26.4049
S8	67	22.8091	S33	11	28.9602	S35	90	18.9533	S11	54	27.2688	S25	31	26.2223
S8	68	22.5136	S33	12	28.9378	S35	91	18.9309	S11	55	27.2177	S25	32	26.0173
S8	69	23.0233	S33	13	28.9067	S35	92	18.8566	S11	56	27.1953	S25	33	26.3229
S8	70	23.004	S33	14	28.9284	S35	93	18.6586	S11	57	27.0649	S25	34	26.2721
S8	71	22.9669	S33	15	28.4652	S35	94	18.5149	S11	58	27.0871	S25	35	24.8069

S8	72	22.8579	S33	16	28.8507	S35	95	18.4841	S11	59	26.9996	S25	36	24.6694
S8	73	21.7231	S33	17	27.872	S35	96	18.2981	S11	60	26.8704	S25	37	25.4241
S8	74	21.6575	S33	18	27.7628	S35	97	18.4302	S11	61	26.8213	S25	38	25.1635
S8	75	22.6522	S33	19	28.2256	S35	98	18.0531	S11	62	26.4345	S25	39	24.9125
S8	76	22.6312	S33	20	27.3591	S35	99	18.0304	S11	63	26.2468	S25	40	24.1717
S8	77	21.2526	S33	21	27.1427	S35	100	17.8322	S11	64	26.0368	S25	41	24.7927
S8	78	22.3895	S33	22	27.7835	S35	101	17.7514	S11	65	25.6292	S25	42	24.0676
S8	79	21.8816	S33	23	27.5991	S35	102	17.716	S11	66	25.4207	S25	43	24.648
S8	80	21.1333	S33	24	26.286	S35	103	17.7105	S11	67	25.3187	S25	44	24.4675
S8	81	21.5204	S33	25	27.3198	S35	104	17.6752	S11	68	24.8479	S25	45	24.4027
S8	82	21.36	S33	26	27.2494	S35	105	17.5161	S11	69	24.7883	S25	46	23.0213
S8	83	21.3481	S33	27	25.1498	S35	106	17.4981	S11	70	24.6419	S25	47	22.9223
S8	84	21.1769	S33	28	26.8017	S35	107	17.4761	S11	71	24.5493	S25	48	22.7204
S8	85	21.1165	S33	29	26.5109	S35	108	17.4313	S11	72	24.3985	S25	49	22.4429
S8	86	21.0827	S33	30	24.817	S35	109	17.3497	S11	73	24.0726	S25	50	24.0463
S8	87	21.0266	S33	31	25.509	S35	110	17.3227	S11	74	23.7983	S25	51	23.9627
S8	88	20.0704	S33	32	24.6147	S35	111	17.2507	S11	75	23.637	S25	52	24.1789
S8	89	20.8408	S33	33	25.2516	S35	112	17.25	S11	76	22.8461	S25	53	21.9903
S8	90	19.9161	S33	34	24.3707	S35	113	17.2264	S11	77	22.4747	S25	54	24.0206
S8	91	20.6734	S33	35	25.0001	S35	114	17.0855	S11	78	21.2502	S25	55	21.7933
S8	92	20.6212	S33	36	24.8467	S35	115	17.0717	S26	1	29.1496	S25	56	21.6343
S8	93	20.5727	S33	37	24.7738	S35	116	17.0315	S26	2	29.1556	S25	57	21.5493
S8	94	19.6862	S33	38	23.3305	S35	117	17.0141	S26	3	29.1432	S25	58	21.509
S8	95	20.2588	S33	39	23.2145	S35	118	17.0143	S26	4	29.1526	S25	59	22.9611
S8	96	19.5817	S33	40	23.0862	S35	119	17.0119	S26	5	29.1451	S25	60	21.4777
S8	97	19.9905	S33	41	22.9745	S35	120	17.0131	S26	6	29.1214	S25	61	22.6791
S8	98	19.9475	S33	42	22.8685	S35	121	17.0032	S26	7	28.9752	S25	62	21.2781
S8	99	19.8348	S33	43	23.3519	S35	122	16.9635	S26	8	28.4696	S25	63	22.3087
S8	100	19.1396	S33	44	23.2735	S35	123	16.9549	S26	9	28.3691	S25	64	22.1782
S8	101	19.6315	S33	45	22.5371	S35	124	16.9045	S26	10	27.9492	S25	65	22.0062
S8	102	19.0011	S33	46	22.9615	S35	125	16.8793	S26	11	26.7854	S25	66	21.9912
S8	103	18.9062	S33	47	22.465	S35	126	16.7233	S26	12	26.621	S25	67	21.9695
S8	104	19.4796	S33	48	22.2948	S35	127	16.8503	S26	13	26.5417	S25	68	21.7838
S8	105	18.7739	S33	49	22.2262	S35	128	16.751	S26	14	26.5149	S25	69	20.8611
S8	106	18.6877	S33	50	22.6033	S35	129	16.701	S26	15	26.4491	S25	70	20.8329
S8	107	18.5864	S33	51	22.1333	S35	130	16.3395	S26	16	26.4199	S25	71	21.5276
S8	108	19.2803	S33	52	22.5473	S35	131	16.5108	S26	17	26.3846	S25	72	21.4951
S8	109	18.3176	S33	53	22.5068	S35	132	16.2594	S26	18	26.3112	S25	73	20.5784
S8	110	18.2316	S33	54	22.4398	S35	133	16.2754	S26	19	26.2721	S25	74	21.5482
S8	111	18.9517	S33	55	21.637	S35	134	16.168	S26	20	26.235	S25	75	20.4353
S8	112	18.1647	S33	56	21.5531	S35	135	16.2264	S26	21	26.2317	S25	76	21.4963
S8	113	18.8606	S33	57	21.5032	S35	136	15.97	S26	22	26.2063	S25	77	20.2755
S8	114	17.8411	S33	58	21.48	S35	137	15.8485	S26	23	26.2188	S25	78	21.3016

S8	115	17.7387	S33	59	21.7575	S35	138	15.7401	S26	24	26.1163	S25	79	20.2354
S8	116	18.5899	S33	60	21.685	S35	139	15.6824	S26	25	26.0641	S25	80	20.2112
S8	117	17.6442	S33	61	21.578	S35	140	15.6369	S26	26	26.0978	S25	81	20.1895
S8	118	17.4856	S33	62	21.0886	S35	141	15.5776	S26	27	26.0383	S25	82	21.0443
S8	119	18.2719	S33	63	21.5287	S31	1	28.5509	S26	28	25.9423	S25	83	20.9047
S8	120	18.1011	S33	64	21.2253	S31	2	28.8081	S26	29	25.8272	S25	84	20.1409
S8	121	18.0194	S33	65	21.0168	S31	3	28.4881	S26	30	25.8041	S25	85	20.8203
S8	122	17.9659	S33	66	21.0651	S31	4	28.4875	S26	31	25.8278	S25	86	20.1094
S8	123	17.092	S33	67	20.9746	S31	5	27.8925	S26	32	25.699	S25	87	19.9781
S8	124	17.0899	S33	68	20.9857	S31	6	28.0102	S26	33	25.5551	S25	88	19.9134
S8	125	17.5587	S33	69	20.974	S31	7	27.4168	S26	34	25.4464	S25	89	20.2799
S8	126	17.0871	S33	70	20.9677	S31	8	27.2282	S26	35	25.4333	S25	90	20.2396
S8	127	17.3299	S33	71	20.8276	S31	9	27.3868	S26	36	25.3773	S25	91	20.2141
S8	128	17.1097	S33	72	20.6839	S31	10	26.5489	S26	37	25.2832	S25	92	19.6157
S8	129	17.0077	S33	73	20.5507	S31	11	26.2468	S26	38	24.9974	S25	93	19.5561
S8	130	16.9366	S33	74	20.3651	S31	12	26.0365	S26	39	25.3409	S25	94	19.518
S8	131	17.0555	S33	75	20.2035	S31	13	26.3699	S26	40	24.8202	S25	95	19.4191
S8	132	16.964	S33	76	19.9478	S31	14	26.2659	S26	41	24.1195	S25	96	19.2702
S8	133	16.8646	S33	77	19.7153	S31	15	26.0298	S26	42	23.8371	S25	97	19.2232
S8	134	16.8299	S33	78	19.5762	S31	16	25.4453	S26	43	23.8865	S25	98	19.8433
S8	135	16.9336	S33	79	19.4273	S31	17	25.665	S26	44	23.8047	S25	99	19.7812
S8	136	16.9245	S33	80	19.1981	S31	18	25.0214	S26	45	23.7107	S25	100	18.9933
S8	137	16.6408	S33	81	19.1176	S31	19	24.596	S26	46	23.6446	S25	101	19.5513
S8	138	16.6265	S33	82	19.184	S31	20	25.1925	S26	47	23.5744	S25	102	18.4892
S8	139	16.6203	S33	83	18.9541	S31	21	24.7856	S26	48	23.4764	S25	103	18.4285
S8	140	16.7127	S33	84	18.905	S31	22	24.2702	S26	49	23.3533	S25	104	18.3682
S8	141	16.7192	S33	85	18.8255	S31	23	24.4446	S26	50	22.91	S25	105	18.3348
S8	142	16.6707	S33	86	18.8576	S31	24	24.3605	S26	51	22.6373	S25	106	18.2978
S8	143	16.5281	S33	87	18.7944	S31	25	23.9864	S26	52	22.6181	S25	107	18.2786
S8	144	16.6273	S33	88	18.7395	S31	26	23.8588	S26	53	22.6099	S25	108	18.4613
S8	145	16.5786	S33	89	18.6799	S31	27	24.089	S26	54	22.597	S25	109	18.3729
S8	146	16.3118	S33	90	18.6356	S31	28	23.7351	S26	55	22.572	S25	110	18.0952
S8	147	16.2808	S33	91	18.624	S31	29	23.918	S26	56	22.2154	S25	111	18.069
S8	148	16.377	S33	92	18.5924	S31	30	23.6263	S26	57	22.1165	S25	112	18.0354
S8	149	16.2341	S33	93	18.5673	S31	31	23.5728	S26	58	22.0455	S25	113	18.0048
S8	150	16.2987	S33	94	18.5502	S31	32	23.5272	S26	59	21.9394	S25	114	17.9792
S8	151	16.2431	S33	95	18.5265	S31	33	23.4368	S26	60	21.8879	S25	115	17.959
S8	152	16.1923	S33	96	18.5129	S31	34	23.7465	S26	61	21.8493	S25	116	17.9375
S8	153	16.1477	S33	97	18.4566	S31	35	23.7289	S26	62	21.8612	S25	117	17.9797
S8	154	16.089	S33	98	18.3956	S31	36	22.9374	S26	63	21.6729	S25	118	17.9512
S8	155	16.0158	S33	99	18.3614	S31	37	22.7973	S26	64	21.5812	S25	119	17.9344
S8	156	15.9937	S33	100	18.3455	S31	38	22.6948	S26	65	21.6836	S25	120	17.6583
S8	157	15.9698	S33	101	18.1856	S31	39	22.6363	S26	66	21.2137	S25	121	17.4861

S8	158	16.0007	S33	102	18.1564	S31	40	23.3561	S26	67	21.1155	S25	122	17.362
S8	159	15.9728	S33	103	18.1368	S31	41	23.1717	S26	68	21.1807	S25	123	17.4566
S8	160	15.8983	S33	104	18.0065	S31	42	23.0281	S26	69	21.124	S25	124	16.9844
S8	161	15.8297	S33	105	17.9672	S31	43	22.9114	S26	70	20.5875	S25	125	16.9377
S8	162	15.8254	S33	106	17.9402	S31	44	22.8412	S26	71	20.8916	S25	126	16.9151
S8	163	15.7683	S33	107	17.8298	S31	45	22.6933	S26	72	20.7642	S25	127	16.8849
S8	164	15.69	S33	108	17.7044	S31	46	21.684	S26	73	20.2597	S25	128	16.9037
S8	165	15.6593	S33	109	17.6495	S31	47	21.5366	S26	74	20.2017	S25	129	16.8858
S8	166	15.6742	S33	110	17.6013	S31	48	21.4491	S26	75	20.4302	S25	130	16.8684
S8	167	15.5951	S33	111	17.5345	S31	49	22.4512	S26	76	20.1435	S25	131	16.8663
S8	168	15.6464	S33	112	17.4881	S31	50	22.368	S27	1	29.28	S25	132	16.8568
S8	169	15.6231	S33	113	17.4575	S31	51	22.2537	S27	2	29.1153	S25	133	16.86
S8	170	15.4475	S33	114	17.2761	S31	52	21.167	S27	3	29.2348	S25	134	16.8473
S8	171	15.4269	S33	115	17.2457	S31	53	21.9255	S27	4	29.2127	S25	135	16.8462
S8	172	15.4923	S33	116	17.2012	S31	54	20.8951	S27	5	29.1781	S25	136	16.8436
S8	173	15.4838	S33	117	17.1915	S31	55	21.7058	S27	6	29.2135	S25	137	16.8428
S8	174	15.4733	S33	118	17.1185	S31	56	20.7496	S27	7	29.1974	S25	138	16.8429
S8	175	15.4674	S33	119	17.0481	S31	57	20.7117	S27	8	29.1847	S25	139	16.8448
S8	176	15.1052	S33	120	17.0292	S31	58	21.3199	S27	9	29.0682	S9	1	29.5306
S8	177	15.3867	S33	121	17.0132	S31	59	20.3526	S27	10	29.0019	S9	2	29.5358
S8	178	15.3586	S33	122	16.9933	S31	60	21.1585	S27	11	28.8344	S9	3	29.5435
S8	179	15.0444	S33	123	16.9488	S31	61	21.1473	S27	12	29.0524	S9	4	29.5326
S8	180	15.3229	S33	124	16.9398	S31	62	20.198	S27	13	29.0089	S9	5	29.5332
S8	181	15.3143	S33	125	16.9211	S31	63	20.8297	S27	14	28.2818	S9	6	29.539
S8	182	14.9893	S33	126	16.8948	S31	64	20.1317	S27	15	28.8819	S9	7	29.526
S8	183	15.1866	S33	127	16.8389	S31	65	19.9879	S27	16	28.7851	S9	8	29.5476
S8	184	15.1415	S33	128	16.8124	S31	66	19.8346	S27	17	28.6459	S9	9	29.5464
S8	185	14.8571	S33	129	16.8077	S31	67	19.782	S27	18	27.9242	S9	10	29.4752
S8	186	14.7865	S33	130	16.7867	S31	68	20.2167	S27	19	27.8722	S9	11	29.3695
S8	187	15.1023	S33	131	16.7915	S31	69	19.6526	S27	20	28.2685	S9	12	29.3727
S8	188	15.092	S33	132	16.7795	S31	70	19.6284	S27	21	28.0784	S9	13	29.3696
S8	189	14.6661	S34	1	29.4964	S31	71	20.1399	S27	22	26.8478	S9	14	29.3953
S8	190	14.9473	S34	2	29.5059	S31	72	19.9745	S27	23	27.9141	S9	15	29.418
S8	191	14.6194	S34	3	29.5221	S31	73	19.2827	S27	24	27.5987	S9	16	29.3949
S8	192	14.8146	S34	4	29.5126	S31	74	19.6926	S27	25	27.2186	S9	17	29.385
S8	193	14.5754	S34	5	29.498	S31	75	19.2417	S27	26	26.7265	S9	18	29.407
S8	194	14.7082	S34	6	29.4377	S31	76	19.5248	S27	27	26.4772	S9	19	28.9631
S8	195	14.6742	S34	7	29.4892	S31	77	19.087	S27	28	26.3749	S9	20	28.9583
S8	196	14.5391	S34	8	29.4631	S31	78	19.0277	S27	29	26.299	S9	21	28.9721
S8	197	14.519	S34	9	29.2039	S31	79	19.0081	S27	30	25.9134	S9	22	28.8492
S8	198	14.5094	S34	10	28.8582	S31	80	18.997	S27	31	26.0806	S9	23	29.2383
S8	199	14.4856	S34	11	28.5544	S31	81	18.9724	S27	32	25.9717	S9	24	29.1381
S8	200	14.4174	S34	12	28.8665	S31	82	18.9981	S27	33	25.8654	S9	25	28.2957

S34	71	20.1707	S34	13	28.0272	S31	83	18.5497	S27	34	25.8319	S9	26	28.3194
S34	72	20.1172	S34	14	28.3518	S31	84	18.4594	S27	35	25.8152	S9	27	28.6295
S34	73	19.4536	S34	15	28.1343	S31	85	18.424	S27	36	25.8047	S9	28	28.2803
S34	74	19.345	S34	16	27.866	S31	86	18.413	S27	37	25.7712	S9	29	28.3511
S34	75	19.6286	S34	17	27.0052	S31	87	18.4061	S27	38	25.7371	S9	30	28.1154
S34	76	19.5654	S34	18	26.8951	S31	88	18.4046	S27	39	25.6745	S9	31	28.3019
S34	77	19.4548	S34	19	26.218	S31	89	18.3979	S27	40	25.1936	S9	32	28.2739
S34	78	19.3305	S34	20	26.854	S31	90	18.3901	S27	41	24.9777	S9	33	28.2094
S34	79	19.0213	S34	21	26.4796	S31	91	18.389	S27	42	24.9332	S9	34	28.1204
S34	80	19.1547	S34	22	26.0415	S32	0	29.4651	S27	43	24.8067	S9	35	27.5727
S34	81	19.0045	S34	23	25.5751	S32	1	29.394	S27	44	24.1642	S9	36	27.5464
S34	82	18.6863	S34	24	25.6721	S32	2	29.4083	S27	45	24.0699	S9	37	27.7932
S34	83	18.6249	S34	25	25.4221	S32	3	29.4485	S27	46	23.9146	S9	38	27.4618
S34	84	18.607	S34	26	25.5178	S32	4	29.4526	S27	47	23.9124	S9	39	27.5926
S34	85	18.5599	S34	27	25.4545	S32	5	29.417	S27	48	23.4801	S9	40	27.4376
S34	86	18.5751	S34	28	25.3418	S32	6	29.4021	S27	49	23.1384	S9	41	27.5014
S34	87	18.46	S34	29	25.2669	S32	7	29.3547	S27	50	22.7604	S9	42	27.441
S34	88	18.4701	S34	30	25.2866	S32	8	29.1339	S27	51	22.4406	S9	43	27.231
S34	89	18.4643	S34	31	25.2829	S32	9	28.9326	S27	52	22.3465	S9	44	27.1029
S34	90	18.4608	S34	32	23.8128	S32	10	28.8586	S27	53	22.0492	S9	45	27.0223
S34	91	18.4532	S34	33	23.7536	S32	11	28.7819	S27	54	21.7779	S9	46	26.7048
S34	92	18.2318	S34	34	23.6729	S32	12	28.7762	S27	55	21.6877	S9	47	26.5507
S34	93	18.2267	S34	35	24.5356	S32	13	28.7495	S27	56	21.7369	S9	48	26.5074
S34	94	18.2139	S34	36	23.999	S32	14	28.7255	S27	57	21.6638	S9	49	26.4908
S34	95	18.1684	S34	37	23.4075	S32	15	28.5612	S27	58	21.5246	S9	50	26.4284
S34	96	18.1212	S34	38	23.3243	S32	16	28.6059	S27	59	21.4535	S9	51	26.4235
S34	97	18.066	S34	39	23.6617	S32	17	28.4892	S27	60	20.9432	S9	52	26.2531
S34	98	18.0261	S34	40	23.1304	S32	18	28.3948	S27	61	20.8244	S9	53	26.2144
S34	99	18.0122	S34	41	22.9508	S32	19	28.0297	S27	62	20.6559	S9	54	26.1855
S34	100	17.9989	S34	42	23.4194	S32	20	27.6077	S27	63	20.6887	S9	55	26.1384
S34	101	17.9025	S34	43	23.3528	S32	21	27.4536	S27	64	20.5708	S9	56	26.2
S34	102	17.8756	S34	44	23.2918	S32	22	27.5358	S27	65	20.3915	S9	57	26.1781
S34	103	17.8511	S34	45	22.4396	S32	23	27.2936	S27	66	20.2239	S9	58	26.1144
S34	104	17.7212	S34	46	23.1009	S32	24	27.2268	S27	67	20.0131	S9	59	25.8286
S34	105	17.6397	S34	47	22.1522	S32	25	27.1037	S27	68	19.9219	S9	60	25.1411
S34	106	17.5225	S34	48	22.0962	S32	26	26.88	S27	69	19.8516	S9	61	24.4363
S34	107	17.5139	S34	49	22.0398	S32	27	26.5816	S27	70	19.7489	S9	62	24.0974
S34	108	17.4784	S34	50	22.5233	S32	28	26.3917	S27	71	19.7053	S9	63	23.888
S34	109	17.4729	S34	51	22.3663	S32	29	26.1797	S27	72	19.6345	S9	64	24.4222
S34	110	17.4551	S34	52	21.6162	S32	30	25.8748	S27	73	19.5448	S9	65	23.6274
S34	111	17.394	S34	53	21.3928	S32	31	25.7382	S27	74	19.5088	S9	66	23.3519
S34	112	17.3638	S34	54	21.2832	S32	32	24.9845	S27	75	19.4879	S9	67	23.1687
S34	113	17.3284	S34	55	21.9201	S32	33	24.0426	S27	76	19.4272	S9	68	22.8875

S34	114	17.3023	S34	56	21.8601	S32	34	23.4661	S27	77	19.3797	S9	69	22.7953
S34	115	17.2593	S34	57	21.7371	S32	35	23.3647	S27	78	19.3373	S9	70	23.4805
S34	116	17.2051	S34	58	21.5012	S32	36	23.0314	S27	79	19.3237	S9	71	22.6764
S34	117	17.1668	S34	59	21.3352	S32	37	22.8903	S27	80	19.2559	S9	72	22.5488
S34	118	17.1336	S34	60	20.8942	S32	38	22.7734	S27	81	19.2206	S9	73	22.3024
S34	119	17.0764	S34	61	20.7966	S32	39	22.6907	S27	82	19.2088	S9	74	22.8298
S34	120	17.0181	S34	62	21.1515	S32	40	22.4087	S27	83	19.1866	S9	75	21.8186
S34	121	16.918	S34	63	21.0551	S32	41	22.0313	S27	84	19.183	S9	76	21.6024
S34	122	16.9068	S34	64	21.0278	S32	42	21.9368	S27	85	19.1048	S9	77	22.6319
S34	123	16.8341	S34	65	20.3827	S32	43	21.798	S27	86	19.0951	S9	78	22.5454
S34	124	16.7531	S34	66	20.8839	S32	44	21.732	S27	87	19.097	S9	79	21.0988
S34	125	16.7231	S34	67	20.7167	S32	45	21.688	S27	88	19.0742	S9	80	20.9752
S34	126	16.6882	S34	68	20.0961	S32	46	21.6308	S27	89	19.018	S9	81	21.7084
S34	127	16.6434	S34	69	19.7921	S32	47	21.616	S27	90	18.9353	S9	82	20.6638
S34	128	16.6106	S34	70	20.2932	S32	48	21.5693	S27	91	18.9393	S9	83	21.4686
S34	129	16.5949				S32	49	21.5556	S27	92	18.8951	S9	84	21.0712
						S32	50	21.4903	S27	93	18.8025	S9	85	20.9779
						S32	51	21.2412	S27	94	18.8361	S9	86	20.4431
						S32	52	21.0381	S27	95	18.7818	S9	87	20.4334
						S32	53	20.9449	S27	96	18.7471	S9	88	20.4286
						S32	54	20.7888	S27	97	18.7287	S9	89	20.5307
						S32	55	20.768	S27	98	18.5447	S9	90	20.5109
						S32	56	20.6346	S27	99	18.4966	S9	91	20.3027
						S32	57	20.4856	S27	100	18.4631	S9	92	20.2476
						S32	58	20.4499	S27	101	18.4291	S9	93	20.1498
						S32	59	20.4053	S27	102	18.4405	S9	94	20.4532
						S32	60	20.4246	S27	103	18.2587	S9	95	20.3427
						S32	61	20.2569	S27	104	18.2237	S9	96	19.929
						S32	62	20.0496	S27	105	18.0118	S9	97	20.1967
						S32	63	19.9198	S27	106	17.9995	S9	98	19.8461
						S32	64	20.0935	S27	107	17.977	S9	99	19.9872
						S32	65	19.9835	S27	108	17.9709	S9	100	19.7307
						S32	66	19.6527	S27	109	17.9567	S9	101	19.7
						S32	67	19.6074	S27	110	17.9425	S9	102	19.615
						S32	68	19.5405	S9	136	16.9614	S9	103	19.5058
						S32	69	19.5755	S9	137	16.6174	S9	104	19.3434
						S32	70	19.4816	S9	138	16.5398	S9	105	19.7383
						S32	71	19.286	S9	139	16.5732	S9	106	19.1447
						S32	72	19.3028	S9	140	16.5286	S9	107	19.0735
						S32	73	19.2229	S9	141	16.4357	S9	108	19.4885
						S32	74	19.1464	S9	142	16.3979	S9	109	18.9619
						S32	75	19.0833	S9	143	16.427	S9	110	19.1692
						S32	76	19.0766	S9	144	16.3799	S9	111	18.6549

						S32	77	19.0368	S9	145	16.3536	S9	112	19.0097
						S32	78	19.0946	S9	146	16.3593	S9	113	18.919
						S32	79	18.8193	S9	147	16.3478	S9	114	18.3084
						S32	80	18.9785	S9	148	16.3325	S9	115	18.0162
						S32	81	18.7365	S9	149	16.3353	S9	116	17.9886
						S32	82	18.7375	S9	150	16.3211	S9	117	18.2581
						S32	83	18.5059	S9	151	16.3209	S9	118	18.1161
						S32	84	18.5566	S9	152	16.3235	S9	119	17.9239
						S32	85	18.4247	S9	153	16.3189	S9	120	17.8573
						S32	86	18.4523	S9	154	16.3185	S9	121	17.8148
						S32	87	18.4246	S9	155	16.2699	S9	122	17.878
						S32	88	18.4023	S9	156	16.2465	S9	123	17.7292
						S32	89	18.3728	S9	157	16.2213	S9	124	17.7837
						S32	90	18.3605	S9	158	16.1979	S9	125	17.648
						S32	91	18.3618	S9	159	16.1713	S9	126	17.6953
						S32	92	18.3505	S9	160	16.1601	S9	127	17.6439
						S32	93	18.3436	S9	161	16.1415	S9	128	17.5228
						S32	94	18.3191	S9	162	16.1123	S9	129	17.483
						S32	95	18.2921	S9	163	16.1031	S9	130	17.5874
						S32	96	18.3145	S9	164	16.0931	S9	131	17.3592
						S32	97	18.279	S9	165	16.0841	S9	132	17.4576
						S32	98	18.0633	S9	166	16.0751	S9	133	17.3541
						S32	99	18.0616	S9	167	16.0637	S9	134	17.1188
									S9	168	16.0433	S9	135	16.8181
									S9	169	16.0365			
									S9	170	16.0401			
									S9	171	16.0291			
