# Supplementary material for: Spatial distribution of the summer subsurface chlorophyll maximum in the North South China Sea
Source: PLoS One. 2021 Apr 7;16(4):e0248715. doi: 10.1371/journal.pone.0248715 (PMC8026054; doi:10.1371/journal.pone.0248715)
Supplement: S4 Fig — (PDF) [file pone.0248715.s004.pdf]

S4 Fig . Vertical profiles of DIN along the six sections shown in the Fig 1

Station	Depth （m）	DIN（μM）	Station	Depth （m）	DIN（μM）
1	2	1.31372857142857	25	75	3.49273571428571
1	17	3.83658571428571	25	100	7.22737472527473
1	30	4.96416483516484	25	138	9.61964670329670
2	2	0.29237032967033	26	2	0.28637582417582
2	25	0.38042142857143	26	15	0.30155769230769
2	50	4.38837307692308	26	25	0.29628846153846
2	60	5.89381098901099	26	48	0.99625521978022
3	2	0.25540989010989	26	75	6.94777197802198
3	25	0.30174835164835	26	100	10.75766208791210
3	55	0.94642005494506	26	120	10.55966593406590
3	75	6.43599725274725	27	2	0.22428626373626
3	89	7.51091153846154	27	15	0.19543956043956
4	2	0.28239725274725	27	25	0.22339120879121
4	25	0.31029615384615	27	50	1.47205631868132
4	50	0.54253076923077	27	75	8.57930329670330
4	70	0.41481263736264	27	100	9.95834285714286
4	105	7.86441538461539	28	2	0.19801538461539
5	2	0.23071868131868	28	15	0.18891923076923
5	25	0.19387637362637	28	25	0.22253296703297
5	44	0.77472087912088	28	45	1.91450631868132
5	75	7.28619120879121	28	75	5.65201813186813
5	100	8.93057637362637	28	89	10.00275164835160
5	130	8.79195494505495	29	2	0.27384945054945
6	2	0.23671428571429	29	15	0.21756648351648
6	25	0.21170494505495	29	25	0.22115934065934
6	59	2.11128956043956	29	31	0.92716703296703
6	75	4.63115714285714	29	50	6.13999890109890
6	100	6.71246703296703	29	60	8.31183901098901
6	150	11.29712637362640	30	2	0.28150604395604
6	200	16.34478076923080	30	15	0.25649505494506
6	300	22.53131923076920	30	25	0.21992252747253

6	365	20.31808021978020	30	44	4.42582060439560
7	2	0.21689780219780	30	80	9.59263791208791
7	25	0.21262032967033	31	2	0.28003461538462
7	55	0.58450631868132	31	15	0.25019780219780
7	75	1.66723681318681	31	25	0.26526868131868
7	100	4.42673351648352	31	61	6.73138434065934
7	150	11.84548736263740	31	75	8.90943516483516
7	200	15.95826923076920	31	90	11.55101263736260
7	300	24.68688571428570	32	2	0.29246978021978
7	500	30.29612362637360	32	25	0.22529945054945
7	645	32.33325604395600	32	50	3.52869175824176
8	2	0.26609285714286	32	75	8.63307032967033
8	25	0.27410164835165	32	100	10.55182142857140
8	50	0.31646263736264	33	2	0.34167417582418
8	75	5.35837692307692	33	15	0.33686538461539
8	100	9.97263901098901	33	25	0.24129340659341
8	150	13.59661593406590	33	55	1.17547060439560
8	200	17.41102307692310	33	75	6.21483241758242
8	300	25.85000934065930	33	100	10.44700879120880
8	500	31.24065824175820	33	135	12.47463461538460
8	800	33.94474340659340	34	2	0.32974945054945
8	959	36.32961098901100	34	25	0.20641978021978
9	2	0.50498021978022	34	56	3.71572197802198
9	25	0.21798351648352	34	75	9.12819450549451
9	50	0.31889065934066	34	100	9.89877087912088
9	63	0.35779670329670	34	159	13.41227362637360
9	100	7.37336318681319	35	2	0.22336208791209
9	150	11.19532197802200	35	25	0.27795659340659
9	172	13.23330879120880	35	50	0.30238956043956
10	2	0.24594120879121	35	65	1.58465659340659
10	25	0.25351263736264	35	100	10.49730384615380
10	56	0.29899642857143	35	150	15.25479285714290
10	75	2.24633846153846	35	188	15.69338956043960
10	100	8.84345989010989	36	2	0.23539956043956
11	2	0.24160219780220	36	25	0.23362560439560
11	25	0.26468406593407	36	50	0.27444428571429
11	50	0.30758516483517	36	68	0.57485527472528
11	70	0.47082609890110	36	100	9.00689835164835

11	78	4.67888791208791	36	150	13.53320219780220
12	2	0.22183571428571	36	200	18.11773956043960
12	25	0.30894065934066	36	300	18.89642032967030
12	50	2.99664285714286	36	500	27.63358626373630
12	75	6.07404340659341	36	800	33.17689285714290
13	2	0.23670000000000	36	1000	37.43875494505490
13	25	0.22081263736264	36	1250	39.99449505494510
13	40	0.59044752747253	37	2	0.21550131868132
13	50	3.76679835164835	37	25	0.15611032967033
14	2	3.23702417582418	37	50	0.26786923076923
14	10	0.37986703296703	37	65	1.58824945054945
14	25	0.25331428571429	37	100	8.63277912087912
14	36	2.75655549450549	37	150	13.95525384615380
15	2	3.75595714285714	37	200	17.51293296703300
15	10	0.19809230769231	37	300	22.00992197802200
15	25	0.78642967032967	37	500	28.88114835164830
15	30	1.55454175824176	37	800	31.02028076923080
16	2	0.20095164835165	37	1000	27.90827582417580
16	10	0.24032252747253	37	1500	36.01484065934070
16	25	0.30649258241758	37	1700	37.99549780219780
16	50	3.98319230769231	38	2	0.28080142857143
17	2	0.22481648351648	38	25	0.29043340659341
17	15	0.22535109890110	38	60	0.62172417582418
17	25	0.34494560439560	38	75	3.85235164835165
17	50	6.99240109890110	38	100	8.65285274725275
17	75	7.77288956043956	38	150	14.04443791208790
18	2	0.25442252747253	38	200	15.75113406593410
18	15	0.22436373626374	38	300	21.51671098901100
18	25	0.22772197802198	38	500	31.35864285714290
18	50	0.78333978021978	38	800	35.34665054945060
18	80	6.28917857142857	38	1000	35.00569835164840
19	2	0.24540329670330	38	1500	35.34995219780220
19	15	0.24573791208791	39	2	0.25927111111111
19	25	0.23507802197802	39	25	0.22624722222222
19	46	0.96439346153846	39	50	0.26215777777778
19	75	3.57805824175824	39	65	3.05278055555556
19	100	5.30418461538462	39	100	10.10402000000000
19	115	9.08743736263736	39	150	14.28421777777780

20	2	0.39760000000000	39	200	17.47902000000000
20	25	0.24319505494506	39	300	22.34251444444440
20	50	0.33740000000000	39	500	27.53676888888890
20	60	0.49494450549451	39	800	33.51633611111110
20	75	4.21759835164835	39	1000	35.07072611111110
20	100	6.82206758241758	39	1350	36.38191944444440
20	150	12.62075659340660	40	2	0.18031333333333
20	200	14.29909065934070	40	25	0.22569722222222
20	300	20.38250329670330	40	50	0.23567666666667
20	470	29.77261923076920	40	66	4.33616000000000
21	2	0.26082362637363	40	100	7.59277166666667
21	25	0.27823461538462	40	150	13.34468222222220
21	50	0.27735989010989	40	200	16.22342500000000
21	65	0.48471730769231	40	300	25.09239611111110
21	100	9.36445274725275	40	500	27.01491944444440
21	150	12.71733571428570	40	800	34.69605222222220
21	200	17.48248076923080	40	950	33.56307944444440
21	300	21.46837857142860	41	2	0.24381500000000
21	500	30.17881978021980	41	25	0.22182055555556
21	800	31.98006428571430	41	56	0.62913083333333
21	935	32.36000000000000	41	75	6.01266388888889
22	2	0.44143076923077	41	100	10.21604555555560
22	25	0.42995659340659	41	150	11.45475277777780
22	50	0.27023021978022	42	2	0.26567833333333
22	65	2.18557005494505	42	25	0.21906555555556
22	100	12.97793406593410	42	41	1.29070944444444
22	150	12.69091483516480	42	75	8.27074055555556
22	190	15.23140384615380	42	100	10.00868444444440
23	2	0.23825000000000	42	150	12.83551222222220
23	25	0.27141208791209	43	2	0.25870777777778
23	52	1.37605027472527	43	25	0.31012277777778
23	75	8.01732692307692	43	38	0.54193333333333
23	100	9.43110219780220	43	75	8.02098055555556
23	150	14.09908076923080	43	100	9.25391333333333
23	165	15.02823736263740	43	125	10.69283500000000
24	2	0.29414010989011	44	2	0.27659944444444
24	25	0.28236483516484	44	25	0.24922222222222
24	50	0.45840549450550	44	40	0.98732555555556

24	64	4.68308736263736	44	75	10.22681388888890
24	100	9.17091868131868	44	90	10.86003555555560
24	150	12.57546208791210	45	2	0.33818000000000
25	2	0.19355549450550	45	15	0.35485944444444
25	15	0.28449010989011	45	30	1.75471250000000
25	25	0.24733131868132	45	50	6.35567222222222
25	52	0.31790027472528	45	75	9.79106277777778
