# Supplementary material for: Spatial distribution of the summer subsurface chlorophyll maximum in the North South China Sea
Source: PLoS One. 2021 Apr 7;16(4):e0248715. doi: 10.1371/journal.pone.0248715 (PMC8026054; doi:10.1371/journal.pone.0248715)
Supplement: S5 Fig — (PDF) [file pone.0248715.s005.pdf]

S5 Fig .Types of vertical profiles of Chl-a (Chl-a, mg·m^{-3}), DIN (DIN, mg·L^{-1}), Phosphorus(P, mg·L^{-1}), Temperature(T,°C) and Salinity(S,‰) along the typical station.

Station1			Station5			Station14			Station28		
Depth(m)	T(°C)	salinity	Depth(m)	T(°C)	salinity	Depth(m)	T(°C)	salinity	Depth(m)	T(°C)	salinity
0	28.8157	28.8378	1	29.4028	30.7704	1	28.5676	20.0484	1	29.3827	31.4917
1	28.8077	28.8378	4	--	31	2	28.805	--	2	29.439	31.574
2	28.9045	29.9037	5	--	32	3	28.7366	20.776	3	29.4349	31.9718
4	28.9007	31.6394	6	29.4256		4	28.7992	--	4	29.3757	--
5	28.9007	31.8487	8	--	33.4448	5	28.8184	--	5	--	32.3948
6	28.8576	32.2584	10	--	33.6473	6	28.714	22.4339	6	--	33.9426
7	--	32.7487	12	29.3074	--	7	28.7015	33.3714	9	28.7208	33.944
8	--	32.8469	17	--	33.6468	8	28.7301	33.6714	10	28.5208	33.9617
9	28.6929	--	18	29.2644	33.6472	9	28.7457	33.7714	15	28.3605	--
10	--	32.8911	20	--	33.6619	10	28.7235	33.7714	16	28.2789	--
11	--	33.0723	21	--	33.6875	11	28.6791	--	17	28.2158	--
12	27.3463	--	22	--	33.6992	12	28.6253	33.8014	18	28.0492	--
13	--	33.451	25	28.9675	--	13	28.6143	--	19	27.9284	33.9715
14	--	--	26	--	33.8521	14	28.6664	--	20	27.8396	33.979
15	25.8678	33.5615	27	--	33.9108	15	--	33.812	22	--	33.9972
16	--	33.7755	29	27.9761	34.0178	16	--	33.8253	23	--	34.0119
17	24.4447	33.9745	31	--	34.1159	17	--	33.85	24	27.6275	--
18	--	34.0051	33	26.2844	--	18	28.2865	--	25	27.371	34.0252
19	--	34.2305	34	25.8576	--	19	--	33.8588	26	27.2951	34.0308
20	23.1436	34.2468	35	--	34.1902	20	--	33.8888	27	27.0004	34.0647
21	22.9712	34.2813	37	--	34.206	21	--	33.8768	28	26.7457	34.0839
22	--	34.2825	38	--	34.2106	22	--	33.8308	30	26.2997	34.1292
23	22.4628	34.3163	39	24.7069	34.2722	23	--	33.861	31	--	34.172
24	22.3043	34.341	40	24.6033	34.2866	24	26.7739	--	32	25.998	--
25	22.1903	--	41	--	34.2901	25	26.5275	--	33	25.1434	34.2926
26	22.1745	--	42	--	34.319	26	26.0056	34.1062	34	24.8006	34.3404
27	22.161	34.3481	43	--	34.3141	27	24.0564	--	35	24.5426	--
28	22.1554	--	44	--	34.3212	28	23.8242	34.2748	36	24.4688	34.3686
29	22.1516	--	47	--	34.4188	29	22.5154	34.4236	37	24.1208	--
30	22.152	--	48	23.2266	34.4359	30	22.1444	34.4406	38	23.8414	34.4818
31	22.1517	34.3412	50	--	34.4363	31	22.0688	34.397	39	23.6189	--
32	22.1517	34.3412	51	--	34.4383	32	21.9401	34.4035	40	23.3728	--
33	22.1517	34.3412	53	--	34.4641	33	21.9224	34.4046	41	23.1934	--
34	22.1517	34.3412	54	22.7746	34.4695	34			42	22.7476	34.5709
35	22.1517	34.3412	57	22.4689	34.4988	35			43	22.4788	--
			58	22.2362	34.5177	36			44	22.3718	--
			59	22.1015	34.5274	37			45	22.2227	--
			61	21.8754	34.5417				46	22.1405	--

			65	21.5871	34.5513				47	22.0312	--
			70	20.6985	34.5989				48	21.9225	--
			72	20.3761	34.6097				49	21.8378	--
			75	19.9977	--				50	21.7651	--
			80	19.6561	34.6127				51	21.7093	--
			85	19.0561	--				55	21.2593	34.6092
			90	18.7815	34.6145				56	--	34.6321
			91	--	34.6169				58	20.7069	--
			94	18.6727	--				59	20.6498	34.6488
			98	18.619	34.6175				60	--	34.6494
			99	18.6596	34.6198				61	--	34.656
			101	18.6617	34.6224				63	20.2022	34.659
			104	18.6761	--				64	--	34.6637
			105	--	34.6261				65	20.0154	--
			106	--	34.6245				66	19.9093	--
			107	--	34.6163				70	19.635	--
			108	--	34.6241				73	19.3502	--
			109	18.5701	34.624				74	19.3161	--
			110	18.5658	34.6224				75	19.2932	34.66
			111	--	34.6226				76	--	34.6428
			112	--	34.6222				77	--	34.6586
			113	--	34.616				78	--	34.649
			114	--	34.6166				79	19.0873	34.642
			115	18.5651	34.6168				80	--	34.6486
			116	18.5656	34.6167				81	--	34.6556
			117	18.5661	34.6169				82	18.9493	34.6496
			118	18.5354	34.6217				83	18.8433	34.6384
			119	--	34.6172				84	--	34.6358
			120	--	34.6216				85	--	34.646
			121	--	34.6172				86	18.6524	34.624
			122	--	34.6169				87	--	34.6372
			123	18.4689	34.6223				89	18.111	34.6389
			124	--	34.6209						
			125	--	34.6206						
			126	--	34.6176						
			127	--	34.6187						
			128	18.4834	34.6197						
			129	18.5054	34.6187						
