# Supplementary material for: Spatial distribution of the summer subsurface chlorophyll maximum in the North South China Sea
Source: PLoS One. 2021 Apr 7;16(4):e0248715. doi: 10.1371/journal.pone.0248715 (PMC8026054; doi:10.1371/journal.pone.0248715)
Supplement: S6 Fig — a: The relationship between Chl-a (mg·m-3) and Fluorescence (mg·m-3), b: The relationship between Chl-a(0) (mg·m-3) and Depth (m), c: The relationship between Chl-a(0) (mg·m-3) and Chl-a(int) (mg·m-2), d: The relationship between Chl-a(m) (mg·m-3) and Chl-a(int) (mg·m-2). (PDF) [file pone.0248715.s006.pdf]

S6 Fig . a:The relationship between Chl-a ($mg \cdot m^{-3}$) and Fluorescence ($mg \cdot m^{-3}$), b: The relationship between Chl-$a_{(0)}$ ($mg \cdot m^{-3}$) and Depth (m), c:The relationship between Chl-$a_{(0)}$ ($mg \cdot m^{-3}$) and Chl-$a_{(int)}$ ($mg \cdot m^{-2}$), d: The relationship between Chl-$a_{(m)}$ ($mg \cdot m^{-3}$) and Chl-$a_{(int)}$ ($mg \cdot m^{-2}$)

Fig 6a		Fig 6b		Fig 6c		Fig 6d	
Chl-a ($mg \cdot m^{-3}$)	Fluorescence ($mg \cdot m^{-3}$)	Chl-$a_{(0)}$ ($mg \cdot m^{-3}$)	Depth (m)	Chl-$a_{(0)}$ ($mg \cdot m^{-3}$)	Chl-$a_{(int)}$ ($mg \cdot m^{-2}$)	Chl-$a_{(m)}$ ($mg \cdot m^{-3}$)	Chl-$a_{(int)}$ ($mg \cdot m^{-2}$)
0.481190436	0.5572	0.20018	55	0.200175734	35	0.481190436	24.665
0.326035836	0.0688	0.10864	44	0.163898397	25	0.581928203	27.4329571
0.470019805	0.0688	0.08131	59	0.108635456	31.06362725	0.815032947	31.06362725
0.581928203	1.0456	0.07871	75	0.086081761	25.75974831	0.687298214	40.02620224
0.185496048	0.252	0.08608	75	0.094201511	29.92549757	0.450590108	37.50857014
0.186717557	0.0078	0.0942	63	0.089250519	29.61465515	0.342425745	25.75974831
0.443790767	0.0688	0.08925	56	0.076791847	19.39647993	0.391712684	29.92549757
0.614246804	0.4962	0.07679	70	0.14445343	32.92289745	0.606293814	29.61465515
0.165638332	0.0078	0.14445	50	0.184872361	48.25412583	0.627759203	32.92289745
0.815032947	1.2288	0.18487	40	0.258571498	57.62173868	1.176630863	48.25412583
0.687298214	0.8014	0.25857	25	0.13447775	33.8101534	1.410098422	57.62173868
0.345528232	0.1299	0.13448	50	0.125541743	23.24030843	1.074307114	61.36976466
0.415658188	0.4962	0.12554	46	0.148093751	25.45790804	0.581645752	33.8101534
0.450590108	0.0688	0.14809	60	0.071987891	26.47080655	0.449744804	23.24030843
0.342425745	0.4962	0.20952	50	0.088292013	28.58420386	0.540127449	25.45790804
0.091682306	0.0078	0.08289	65	0.09	21.1768784	0.339281773	28.73206787
0.22657286	0.0688	0.07973	52	0.08	25.94828892	0.560053247	37.97971613
0.391712684	0.8014	0.07199	64	0.164397098	23.5938262	0.662137149	45.08515429
0.606293814	0.3741	0.08829	75	0.224712282	29.20445389	0.423031892	26.47080655
0.434004254	0.313	0.07306	48	0.124018225	27.48597887	0.406658609	28.58420386
0.6823405	0.6793	0.08	50	0.09357099	30	0.369648782	21.1768784
0.378661419	0.1299	0.1644	45	0.10090862	33.14987871	0.41	25.94828892
0.60280548	0.4962	0.43602	31	0.111058583	36.35715762	0.316609925	23.5938262
0.627759203	0.6183	0.24507	44	0.061211501	25	0.511384545	29.7674115
0.293294803	0.252	0.12402	50	0.077000407	21.72651799	0.343773872	29.20445389
0.643020388	0.6793	0.09357	55	0.11	26.45347475	0.537554908	27.48597887
1.176630863	1.473	0.10091	56	0.147651567	28.90892638	0.459559367	38.47217784
1.410098422	2.2056	0.11106	65	0.213156871	30.50120699	0.461675783	33.14987871

0.673607078	0.4962	0.06121	60	0.172914105	31.82181317	0.515693175	36.35715762
1.074307114	1.9003	0.077	65	0.16159951	31.40673494	0.256960893	28.83735632
0.565381051	0.4962	0.09339	60	0.280604997	32.63336439	0.458644792	21.72651799
0.581645752	0.6793	0.21316	56			0.499699444	35.47079503
0.449744804	0.3741	0.17291	41			0.558671812	26.45347475
0.232559042	0.0688	0.1616	38			0.393988113	28.90892638
0.322345692	0.1909	0.2806	40			0.396595179	30.50120699
0.540127449	0.7404	0.29144	30			0.332303113	31.82181317
0.251067662	0.0688					0.474107593	31.40673494
0.305526021	0.252					0.597170576	32.63336439
0.130438513	0.0688						
0.560053247	0.7404						
0.662137149	1.0456						
0.222613572	0.3741						
0.423031892	0.9846						
0.235779588	0.1299						
0.406658609	0.0688						
0.369648782	0.4962						
0.257286334	0.1909						
0.41	0.4962						
0.23	0.1909						
0.164397098	0.0688						
0.316609925	0.4351						
0.207175668	0.1299						
0.436024731	0.0078						
0.287120864	0.252						
0.293653136	0.252						
0.511384545	0.8014						
0.284605864	0.1299						
0.558671812	0.5572						
0.33156924	0.0688						
0.393988113	0.4962						
0.153737119	0.0078						
0.313036164	0.0078						
0.396595179	0.313						
0.253882099	0.313						
0.332303113	0.5572						
0.186959508	0.0688						
0.474107593	0.6183						
0.147998724	0.0078						
0.597170576	0.6793						
0.190950305	0.252						
0.588267213	0.5572						

0.30167075	0.313						
0.168831877	0.0078						
0.558671812	0.5572						
0.30167075	0.313						
0.168831877	0.0078						
