# Supplementary material for: Spatial distribution of the summer subsurface chlorophyll maximum in the North South China Sea
Source: PLoS One. 2021 Apr 7;16(4):e0248715. doi: 10.1371/journal.pone.0248715 (PMC8026054; doi:10.1371/journal.pone.0248715)
Supplement: S7 Fig — a: Relationship between In-suit Chl-a (mg·m-3) and MODIS Chl-a (mg·m-3); b: Distribution characteristics of Chl-a in NSCS. (PDF) [file pone.0248715.s007.pdf]

S7 Fig . a: Relationship between In-suit Chl-a (mg·m^{-3}) and MODIS Chl-a (mg·m^{-3}); b: Distribution characteristics of Chl-a in NSCS

Fig 7a	
MODIS Chl-a (mg·m-3)	In-suit Chl-a (mg·m-3)
0.097	0.11
0.089	0.08
0.0698	0.09
0.0835	0.08
0.100525	0.14
0.112225	0.16
0.11485	0.13
0.09115	0.13
0.089925	0.08
0.081275	0.08
0.092925	0.07
0.1176	0.09
0.1024	0.07
0.1639	0.16
0.201975	0.25
0.1589	0.22
0.122	0.12
0.10715	0.09
0.108525	0.10
0.102725	0.11
0.0876	0.06
0.1254	0.08
0.1269	0.09
0.142275	0.19
0.138125	0.15
0.151525	0.17
0.134575	0.16

Fig 7b Data Availability: Chl-a data files is freely available from the GlobColour dataset (https://hermes.acri.fr/).
